# Nanosized Ti-Based Perovskite Oxides as Acid–Base Bifunctional Catalysts for Cyanosilylation of Carbonyl Compounds

**DOI:** 10.1021/acsami.3c01629

**Published:** 2023-04-03

**Authors:** Takeshi Aihara, Wataru Aoki, Shin Kiyohara, Yu Kumagai, Keigo Kamata, Michikazu Hara

**Affiliations:** † Laboratory for Materials and Structures, Institute of Innovative Research, Tokyo Institute of Technology, Nagatsuta-cho 4259, Midori-ku, Yokohama 226-8503, Japan; ‡ Institute for Materials Research, Tohoku University, 2-1-1 Katahira, Aoba-ku, Sendai 980-8577, Japan

**Keywords:** perovskite-type oxides, nanoparticle, strontium titanate, cyanosilylation, acid−base bifunctional catalysts

## Abstract

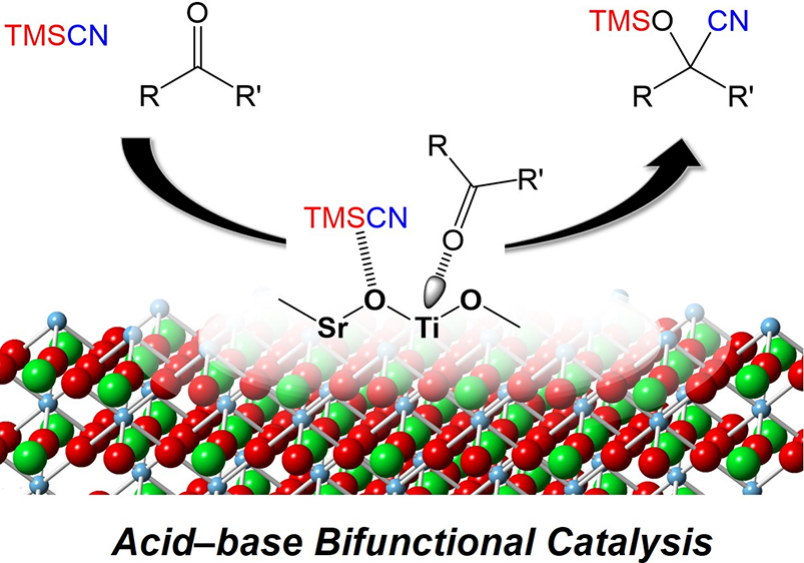

The development of effective solid acid–base bifunctional catalysts remains a challenge because of the difficulty associated with designing and controlling their active sites. In the present study, highly pure perovskite oxide nanoparticles with *d*^0^-transition-metal cations such as Ti^4+^, Zr^4+^, and Nb^5+^ as *B*-site elements were successfully synthesized by a sol–gel method using dicarboxylic acids. Moreover, the specific surface area of SrTiO_3_ was increased to 46 m^2^ g^–1^ by a simple procedure of changing the atmosphere from N_2_ to air during calcination of an amorphous precursor. The resultant SrTiO_3_ nanoparticles showed the highest catalytic activity for the cyanosilylation of acetophenone with trimethylsilyl cyanide (TMSCN) among the tested catalysts not subjected to a thermal pretreatment. Various aromatic and aliphatic carbonyl compounds were efficiently converted to the corresponding cyanohydrin silyl ethers in good-to-excellent yields. The present system was applicable to a larger-scale reaction of acetophenone with TMSCN (10 mmol scale), in which 2.06 g of the analytically pure corresponding product was isolated. In this case, the reaction rate was 8.4 mmol g^–1^ min^–1^, which is the highest rate among those reported for heterogeneous catalyst systems that do not involve a pretreatment. Mechanistic studies, including studies of the catalyst effect, Fourier transform infrared spectroscopy, and temperature-programmed desorption measurements using probe molecules such as pyridine, acetophenone, CO_2_, and CHCl_3_, and the poisoning effect of pyridine and acetic acid toward the cyanosilylation, revealed that moderate-strength acid and base sites present in moderate amounts on SrTiO_3_ most likely enable SrTiO_3_ to act as a bifunctional acid–base solid catalyst through cooperative activation of carbonyl compounds and TMSCN. This bifunctional catalysis through SrTiO_3_ resulted in high catalytic performance even without a heat pretreatment, in sharp contrast to the performance of basic MgO and acidic TiO_2_ catalysts.

## 1 Introduction

Acid and base catalysts are important materials because they are widely used in industrially relevant chemical processes, including petroleum refining, biomass conversion, and fine chemical synthesis.^1−3^ Recently, synergistic and cooperative acid–base catalysis, in which acid and base sites can activate electrophiles and nucleophiles in concert, respectively, has attracted intensive attention.^4−6^ Such acid–base bifunctionalities of metal oxides, zeolites, metal phosphates, supported catalysts, and organocatalysts have led to specific activity and selectivity in various reactions (e.g., hydration,^7,8^ aldol condensation,^9,10^ Knoevenagel condensation,^11,12^ CO_2_ fixation,^13−15^ Meerwein–Ponndorf–Verley reduction,^16,17^ acetalization,^18^ C–H bond activation,^19^ and asymmetric syntheses^20,21^) in comparison with single-function acid or base catalysts. Compared with homogeneous and stoichiometric acid–base catalysts, solid acid–base catalysts offer several advantages, such as recovery/reuse of the catalysts and no formation of waste salts; however, controlling the structure of cooperatively workable active sites on solid catalysts is difficult, which limits their acid–base catalyst performance. Therefore, the design and development of effective solid acid–base bifunctional catalysts are key challenges in catalysis research.

Perovskite-type oxides have emerged as an important class of functional mixed-oxide materials in the fields of magneticity, ferroelectricity, piezoelectricity, and catalysis.^22−24^ Their general formula is *AB*O_3_, where *A* represents an alkali (+1), alkaline-earth (+2), or lanthanide metal (+3) cation and *B* represents a transition-metal cation (+5, +4, or +3). The structures and physiochemical properties of perovskite-type oxides can be tuned by controlling their versatile chemical composition.^25,26^ Catalysis over various perovskite-type oxides has been mainly investigated for electrochemical,^27,28^ photocatalytic,^29,30^ high-temperature gas-phase (e.g., combustion and NO*x* decomposition),^31−33^ and selective oxidation reactions^34,35^ because of their good structural stability, flexibility, and controllability; however, acid–base catalysis using perovskite-type oxides has not been sufficiently explored.^36−38^ Wu and co-workers have reported pioneering studies on the acid–base catalysis of perovskite oxides for several gas-phase reactions, including investigations of their structure–activity relationship; however, the application of perovskite oxides to liquid-phase organic reactions has been limited.^36^ Coprecipitation, sol–gel, solution combustion, and soft/hard templating methods are typically used to synthesize nanosized and/or porous perovskite oxides for use in catalytic applications (Table S1). However, these methods typically involve complicated multistep operations (e.g., pH adjustment or post-treatment), the use of toxic reagents and/or structure-directing agents, and high-temperature calcination, which results in aggregation of particles and a concomitant decrease in the catalyst specific surface area. Therefore, a simple and effective method for synthesizing highly pure perovskite oxides with high specific surface areas is desirable.

**Figure 1:**
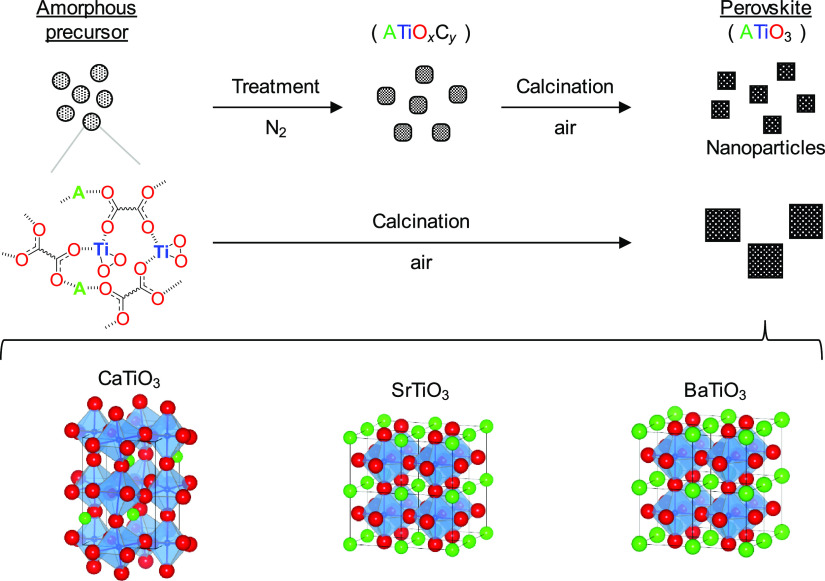
Schematic representations of the sol–gel synthesis method used in present study and the structures of the Ti-based perovskite oxides. Green, blue, and red spheres represent *A*-site metal cations, Ti^4+^ cations, and O^2–^ anions, respectively.

Our group recently reported a facile sol–gel method for preparing various nanosized perovskite oxides (e.g., SrMnO_3_, BaRuO_3_, and BaFeO_3−δ_) using aspartic or malic acid and investigated their catalytic activity toward aerobic oxidation and electrochemical reactions.^39−45^ Because of their unique face-sharing octahedral units in hexagonal structures, these perovskite oxides exhibited remarkable catalytic performance for the selective oxidation of various hydrocarbons and sulfides with O_2_ as the sole oxidant. Our sol–gel method includes the calcination of amorphous precursors prepared via a ligand-exchange reaction of metal acetates and dicarboxylic acids as starting materials; therefore, the method is mainly limited to the synthesis of perovskite oxides with *B*-site metal cations consisting of group 7–10 elements commercially available as acetate salts. Herein, we apply this method to the synthesis of high-surface-area perovskite oxides containing *d*^0^-transition metals (Ti^4+^, Zr^4+^, and Nb^5+^), which are not available as acetate salts, as acid–base solid catalysts. Despite a simple procedure in which the atmosphere is controlled during thermal treatment of amorphous precursors, the specific surface areas of the resultant perovskite oxides and their catalytic activity can be dramatically improved (Figure 1). SrTiO_3_ nanoparticles function as effective reusable solid catalysts for the cyanosilylation of various carbonyl compounds, which is an important C–C bond-forming reaction to produce the corresponding cyanohydrin silyl ethers used as key building blocks for α-hydroxy ketones, *α*-hydroxy acids, and β-amino alcohols,^46,47^ even without a thermal pretreatment. Detailed surface structural analyses reveal the importance of cooperative acid–base catalysis of SrTiO_3_ for achieving high cyanosilylation performance.

## 2 Experimental Section

### 2.1 Instruments

The physicochemical properties of the solid materials were investigated using X-ray diffraction (XRD), nitrogen adsorption–desorption, thermogravimetry–differential thermal analysis (TG–DTA), inductively coupled plasma–atomic emission spectroscopy (ICP–AES), scanning electron microscopy (SEM), transmission electron microscopy (TEM), Fourier transform infrared (FT-IR) spectroscopy, temperature-programmed desorption (TPD) analysis, and X-ray photoelectron spectroscopy (XPS) using previously reported instruments.^40,48^ X-Ray adsorption spectroscopy (XAS) analysis of the catalysts was performed at the BL01B1 beamline at SPring-8 (Japan Synchrotron Radiation Research Institute, Hyogo, Japan). The ring energy was 8 GeV, and the stored current was 99.5 mA. Ti *K*-edge (4.96 keV) X-ray absorption spectra were recorded using an Si(111) double-crystal monochromator. All spectra were recorded using the transmission method in quick-scan mode with ion chambers as detectors. Data reduction was performed using xTunes (Science & Technology Instruments).^49^ Gas chromatography (GC), GC–mass spectrometry (GC–MS), and nuclear magnetic resonance (NMR) analyses of products from the catalytic reactions were performed using previously reported methods.^40^ The details are described in the Supporting Information. The crystal structures in the present work were generated using the VESTA ver. 3.5.6 software.^50,51^

### 2.2 Synthesis of Perovskite Oxides Containing *d*^0^-Transition Metals (Ti^4+^, Zr^4+^, and Nb^5+^)

Perovskite oxides containing *d*^0^-transition metals (Ti^4+^, Zr^4+^, and Nb^5+^) were synthesized by a sol–gel method using dicarboxylic acids such as aspartic and malic acids. A typical procedure for the synthesis of titanates *A*TiO_3_ (*A* = Ca^2+^, Sr^2+^, Ba^2+^) was as follows: first, titanium(IV) isopropoxide (Ti(O*i*-Pr)_4_; 5 mmol) was added dropwise to an aqueous solution (200 mL) containing dl-malic acid (20 mmol) and 30% aqueous H_2_O_2_ (20 mmol). The suspension was vigorously stirred until Ti(O*i*-Pr)_4_ was completely dissolved, followed by the addition of the *A*-site metal acetate (5 mmol). The solution was evaporated to dryness, and the resultant red–orange solid was dried at 463 K for 1 h to give a pale-yellow powder referred to hereafter as the precursor. The obtained precursor was calcined under an (i) N_2_/air or (ii) air atmosphere as follows: (i) the precursor was heated at a rate of 3 K min^–1^ from room temperature to 823 K under an N_2_ atmosphere and then calcined at the corresponding temperature for 5 h under an air atmosphere to give *A*TiO_3__N_2_-air. (ii) The calcination conditions (heating rate, temperature, and time) were the same as in those in case i, but all processes were performed under an air atmosphere. Elemental analysis: calcd (%) for CaTiO_3__N_2_-air: Ca 29.5, Ti 35.2; found: Ca 29.0, Ti 34.6. For SrTiO_3__N_2_-air: Sr 47.8, Ti 26.1; found: Sr 46.2, Ti 25.2. For SrTiO_3__air: Sr 47.8, Ti 26.1; found: Sr 48.2, Ti 25.7. For BaTiO_3__N_2_-air: Ba 58.9, Ti 20.5; found: Ba 61.1, Ti 19.9. Niobates were also obtained via the same procedure using niobium(V) ethoxide (Nb(OEt)_5_), alkali-metal acetates, malic acid (2 equivalents relative to the total metal amounts), and H_2_O_2_ (4 equivalents relative to Nb). Hydrogen peroxide is required to react with Ti and Nb to form water-soluble metal-peroxo species.^52^ Zirconates were synthesized via a H_2_O_2_-free sol–gel method using zirconium oxyacetate (ZrO(OAc)_2_), alkaline-earth-metal acetates, and aspartic acid (1.5 equivalents relative to the total metal amounts). The details are shown in Table 1.

**Table 1:** Structure, Grain Size, and Specific Surface Area of *AB*O_3__air Catalysts

entry	catalyst	calcination temp./K	system	*D*^a^/nm	*S*_BET_^b^/m^2^ g^–1^
1	CaTiO_3_	823	orthorhombic	33 (29)	26 (31)
2	SrTiO_3_	823	cubic	31 (23)	30 (46)
3	BaTiO_3_	823	tetragonal	22 (21)	15 (18)
4	CaZrO_3_	823	orthorhombic	48	9
5	SrZrO_3_	923	orthorhombic	43	4
6	BaZrO_3_	923	cubic	44	16
7	LiNbO_3_	773	trigonal	34	15
8	NaNbO_3_	773	orthorhombic	29	23
9	KNbO_3_	823	orthorhombic	47	4

a Grain size estimated using the Scherrer equation.

b Surface area estimated from N_2_ adsorption. Values of *AB*O_3__N_2_-air are shown in parentheses.

### 2.3 Procedure for Catalytic Cyanosilylation of Carbonyl Compounds with Trimethylsilyl Cyanide

The catalytic cyanosilylation reaction of various carbonyl compounds was conducted in a 20 mL glass vessel containing a magnetic stirring bar. A typical procedure for cyanosilylation was as follows: catalyst (50 mg), carbonyl compound (1 mmol), trimethylsilyl cyanide (TMSCN; 1.5 mmol), toluene (2 mL), and *n*-decane (0.5 mmol) as an internal standard were added to a reactor under an Ar atmosphere. The reaction mixture was stirred in an ice bath (275 K) or an organic synthesizer (298 K) and periodically analyzed using GC with a flame-ionization detector (GC-FID). After the reaction proceeded completely, the catalyst was recovered by filtration. The products were isolated by removing the solvent and TMSCN remaining in the filtrate using a kugelrohr distillation apparatus and were subsequently identified by comparison of their NMR spectra with those previously reported. The recovered catalysts were washed with toluene (25 mL) and MeOH (25 mL), dried at 373 K for 6 h, and then calcined at 823 K for 1 h to remove residues from their surface prior to them being recycled.

### 2.4 Quantum Chemical Calculations

We prepared two slab models with Ti-O- or Sr-O-terminated surfaces by cleaving the bulk material along the (100) plane. After optimizing the pristine surface models, we placed a TMSCN molecule on the planes so that the Si atom was located above an O atom and the cyanogen above a Ti or Sr atom. We then optimized the structures and estimated the adsorption energies. The density functional theory calculations were performed using the projector augmented-wave (PAW) method^53^ as implemented in the VASP code.^54^ We used the Perdew-Burke-Ernzerhof functional tuned for solids (PBEsol)^55^ and employed plane-wave cutoff energies of 520 and 400 eV for the perfect crystal and slab models, respectively. The *k*-point densities were set to be higher than 2.5 and 1.8 Å^–1^ for the bulk and slabs, respectively. We employed PAW data sets with radial cutoffs of 1.32, 1.48, 0.80, 0.79, 1.01, 0.79, and 0.5 Å for Sr, Ti, O, C, Si, N, and H, respectively, and described Sr-4s, 4p, and 5s, Ti-3d and 4s, O-2s and 2p, C-2s and 2p, Si-2s and 2p, N-2s and 2p, and H-1s as valence electrons.

## 3 Results and Discussion

### 3.1 Synthesis and Characterization of Perovskite Oxide Nanoparticles with *d*^0^-Transition Metals as *B*-Site Metal Cations

**Figure 2:**
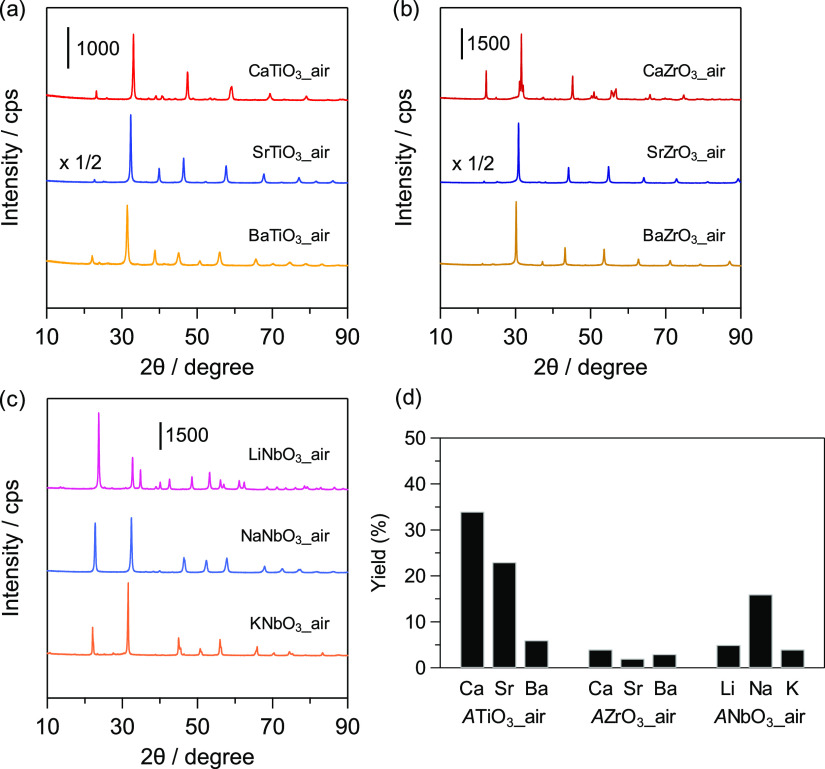
(a–c) XRD patterns for *d*^0^-transition-metal-cation-based perovskite oxides *AB*O_3__air. (d) Cyanosilylation of **1a** with TMSCN over *AB*O_3__air catalysts. Conditions: catalyst (50 mg), **1a** (1.0 mmol), TMSCN (1.5 mmol), toluene (2 mL), ice bath (275 K), 15 min, Ar atmosphere.

Because the preparation of amorphous precursors using dicarboxylic acids with lower carbon contents is a key step in obtaining pure nanosized perovskite oxides at low calcination temperatures,^39−45^ we investigated the solution states of *d*^0^-transition metals (Ti^4+^, Zr^4+^, and Nb^5+^) in the presence of dicarboxylic acids. Soluble Ti^4+^, Zr^4+^, and Nb^5+^ species in water were prepared by mixing Ti(O*i*-Pr)_4_/malic acid/H_2_O_2_, ZrO(OAc)_2_/aspartic acid, and Nb(OEt)_5_/malic acid/H_2_O_2_, respectively. The combination of these aqueous solutions with the corresponding *A*-site metal acetates (*A*: alkali (Li^+^, Na^+^, K^+^) and alkaline-earth (Ca^2+^, Sr^2+^, Ba^2+^) metal cations) in a molar ratio of *A*:*B* = 1:1 gave *AB*O_3_ precursors that were amorphous, as confirmed by the lack of peaks in their XRD patterns (Figures S1 and S2). The calcination of the precursors at appropriate temperatures in air resulted in the formation of analytically pure perovskite oxides (denoted as *AB*O_3__air). Figure 2a shows XRD patterns for the titanates (*A*TiO_3__air: orthorhombic CaTiO_3_, cubic SrTiO_3_, tetragonal BaTiO_3_), zirconates (*A*ZrO_3__air: orthorhombic CaZrO_3_, orthorhombic SrZrO_3_, cubic BaZrO_3_), and niobates (*A*NbO_3__air: trigonal LiNbO_3_, orthorhombic NaNbO_3_, orthorhombic KNbO_3_). Details of the perovskite oxides (i.e., their calcination temperature, specific surface area, and grain size) are summarized in Table 1. Although the specific surface area and grain size differed depending on the material, aggregates of spherical-like nanoparticles were observed in the SEM images, which are similar to the SEM images of nanosized perovskite oxide particles previously synthesized using our sol–gel methods (Figures S3).^39−41^

In addition, the specific surface area of the perovskite oxides was improved by changing the atmosphere during the calcination of the precursors from air to N_2_–air (i.e., an N_2_ atmosphere during heating of the precursor to the target temperature, followed by an air atmosphere during calcination, Figure 1). We investigated the effect of the reaction atmosphere on the synthesis of titanates, which were much more active than zirconates and niobates toward the cyanosilylation of acetophenone (**1a**) with TMSCN, as detailed below (Figure 2d). XRD patterns for the titanates calcined under N_2_–air (denoted as *A*TiO_3__N_2_-air) are shown in Figure 2a and Figure S1. The crystal structure of *A*TiO_3__N_2_-air was the same as that for *A*TiO_3__air despite the different calcination atmospheres. Elemental analysis by ICP–AES indicated that the contents of the *A*-site metal and Ti in the bulk structure of the synthesized titanates were consistent with their theoretical values. However, the specific surface area for *A*TiO_3__N_2_-air (18–46 m^2^ g^–1^) was greater than that for *A*TiO_3__air (15–30 m^2^ g^–1^).

**Figure 3:**
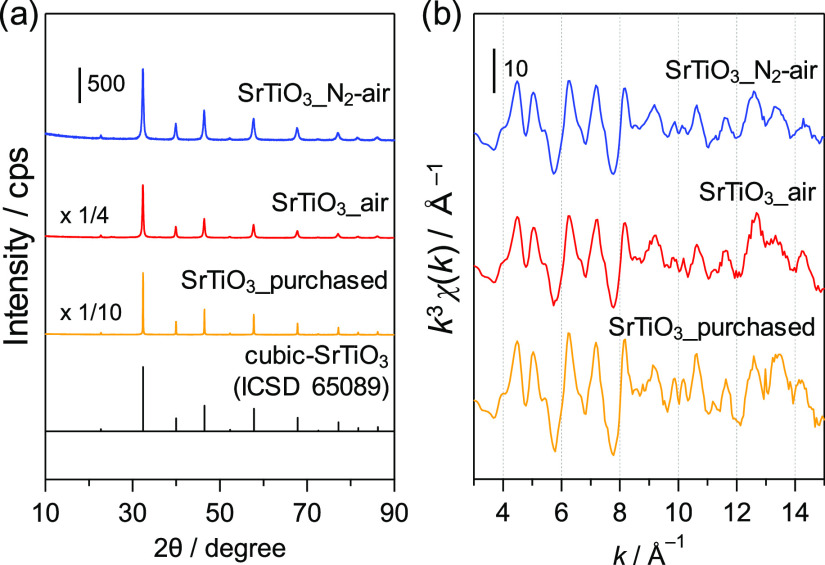
(a) XRD patterns and (b) Ti *K*-edge EXAFS oscillations for SrTiO_3_.

**Figure 4:**
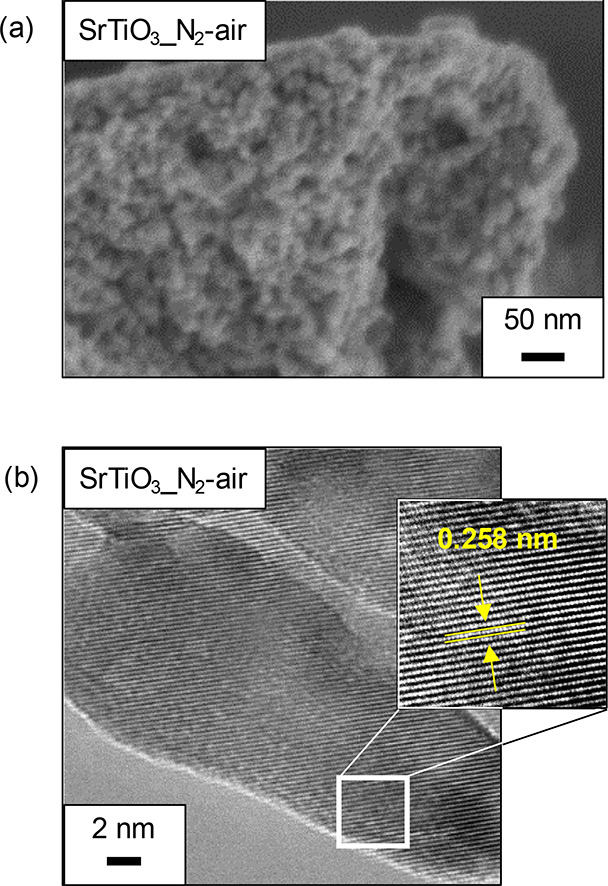
(a) SEM and (b) TEM images of SrTiO_3__N_2_-air.

To investigate the effect of the calcination atmosphere, we analyzed the SrTiO_3_ precursor as a representative titanate under N_2_ and air atmospheres (Figure S4). Two exothermic peaks at 723 and 823 K were observed under an air atmosphere, accompanied by a large weight loss (62%); these peaks are likely attributable to the combustion of organic components in the precursor and to the crystallization of SrTiO_3_, respectively. However, the thermogram corresponding to calcination of the precursor under an N_2_ atmosphere showed no clear exothermic peak despite a weight loss (45 wt %) at ∼823 K. No XRD peaks associated with SrTiO_3_ were observed for the sample obtained by calcination of the precursor at 823 K under an N_2_ atmosphere; thus, crystallization of SrTiO_3_ requires the presence of O_2_ (Figure S1). Such a difference in the decomposition processes of the precursor (i.e., combustion and pyrolysis under oxidative and inert atmospheres) likely affects the growth and aggregation of SrTiO_3_ nanoparticles, whose formation was confirmed by XRD, X-ray absorption fine structure (XAFS), SEM, and TEM analyses. The grain size in SrTiO_3__N_2_-air was estimated using Scherrer’s equation to be 23 nm on the basis of the (110) diffraction peak, which is smaller than the grain size in SrTiO_3__air (31 nm) (Figure 3a). The Ti *K*-edge extended X-ray absorption fine structure (EXAFS) oscillation for each SrTiO_3_ sample was also measured. Although no significant difference of the EXAFS oscillation period was observed among the SrTiO_3_ samples, the peak amplitude for SrTiO_3__N_2_-air was smaller than those for SrTiO_3__air and a purchased SrTiO_3_ sample (Figure 3b and Figure S5). These results suggest that the local structure of Ti is the same for the SrTiO_3__N_2_-air and SrTiO_3__air samples and that the SrTiO_3_ particle size decreased after the N_2_ treatment. The results of these X-ray structural characterizations are in good agreement with the SEM and TEM observations. SEM images of SrTiO_3__N_2_-air and SrTiO_3__air show agglomerates of nanoparticles with a spherical morphology; however, the particle size (approximately 30–40 nm) in SrTiO_3__air is larger than that (approximately 10–30 nm) in SrTiO_3__N_2_-air, likely because of sintering of the nanoparticles in the former case (Figures S3 and 4a). TEM images of SrTiO_3_ are shown in Figure 4b and Figure S6. The images of SrTiO_3_ particles in both the SrTiO_3__N_2_-air and SrTiO_3__air samples clearly display lattice fringes associated with (110) planes, indicating that the synthesized SrTiO_3_ is highly crystalline. The particle size of SrTiO_3__N_2_-air estimated from the BET surface area and density (5.12 cm^3^ g^–1^) assuming that the particles are spherical was 26 nm, which is comparable to the grain size calculated from the (110) diffraction peak in the XRD patterns using Scherrer’s equation (23 nm). SrTiO_3_ nanoparticles were also obtained using Ar instead of N_2_ during calcination (SrTiO_3__Ar-air). The grain size and specific surface area of SrTiO_3__Ar-air were 25 nm and 41 m^2^ g^–1^, respectively, comparable to those of SrTiO_3__N_2_-air. No substantial difference was observed in the XRD patterns or in the catalytic activity between SrTiO_3__N_2_-air and SrTiO_3__Ar-air, which indicates that the type of inert gas does not affect the structure or the catalytic performance of the SrTiO_3_ nanoparticles (Figure S1a,b).

The specific surface area for SrTiO_3__N_2_-air was 46 m^2^ g^–1^, which was much larger than those for SrTiO_3__air (30 m^2^ g^–1^) and the purchased sample (4 m^2^ g^–1^). The literature includes three reports on high-specific-surface-area (>50 m^2^ g^–1^) SrTiO_3_ synthesized by other methods; however, those samples either contained impurities or required a post-treatment to remove coproduced impurities such as SrCO_3_ (Table S1). By contrast, we obtained highly pure SrTiO_3_ with a high specific surface area by only switching the atmosphere from N_2_ to air during the calcination process. Although high-specific-surface-area perovskite oxides have been similarly obtained using a hard template formed *in situ* by carbonization of polymeric carbonaceous precursors at high temperatures (>973 K) under an inert atmosphere,^56,57^ our method does not require a high-temperature treatment, likely because most of the carbon species can be removed from the amorphous precursor by pyrolysis at lower temperatures. The specific surface areas and grain sizes for the other titanates (CaTiO_3_ and BaTiO_3_) showed trends similar to those observed for SrTiO_3_; thus, various titanate nanoparticles with high specific surface areas were successfully synthesized using the proposed method. After calcination of the amorphous precursor prepared using citric acid instead of malic acid under N_2_-air, only the XRD peaks attributable to cubic SrTiO_3_ (SrTiO_3_(CA)_N_2_-air) were observed (Figure S1a and b). The grain size of SrTiO_3_(CA)_N_2_-air was 32 nm, larger than that (23 nm) of SrTiO_3__N_2_-air prepared using malic acid. In addition, the specific surface area of SrTiO_3_(CA)_N_2_-air was 26 m^2^ g^–1^, smaller than that (45 m^2^ g^–1^) of SrTiO_3__N_2_-air. Malic acid has demonstrated similar effectiveness as a chelating reagent in the synthesis of other perovskite oxides.^40,58^

### 3.2 Catalysis of Perovskite Oxides for Cyanosilylation of Carbonyl Compounds

We evaluated the acid–base catalysis of the synthesized perovskite oxides using the cyanosilylation of acetophenone (**1a**) with 1.5 equivalents of TMSCN to form the corresponding cyanohydrin trimethylsilyl ether (**2a**) at 275 K in an ice bath under an Ar atmosphere. The cyanosilylation of carbonyl compounds with silyl cyanides is a reaction well known to be promoted by acid and/or base catalysts.^59,60^ Although effective solid catalysts such as metal-cation-exchanged montmorillonites (Sn- and Fe-Mont), Al-MCM-41, hydroxyapatite, and hydrotalcite have been used for the cyanosilylation of carbonyl compounds with TMSCN (Table S2), most of these heterogeneous systems typically require a thermal pretreatment of the catalyst at 393–453 K under vacuum to remove adsorbed species such as H_2_O, CO_2_, and organic compounds on their active sites.^61,62^ Therefore, the development of a new solid acid–base catalyst that functions efficiently even in the presence of poisoning molecules remains a challenge.

**Figure 5:**
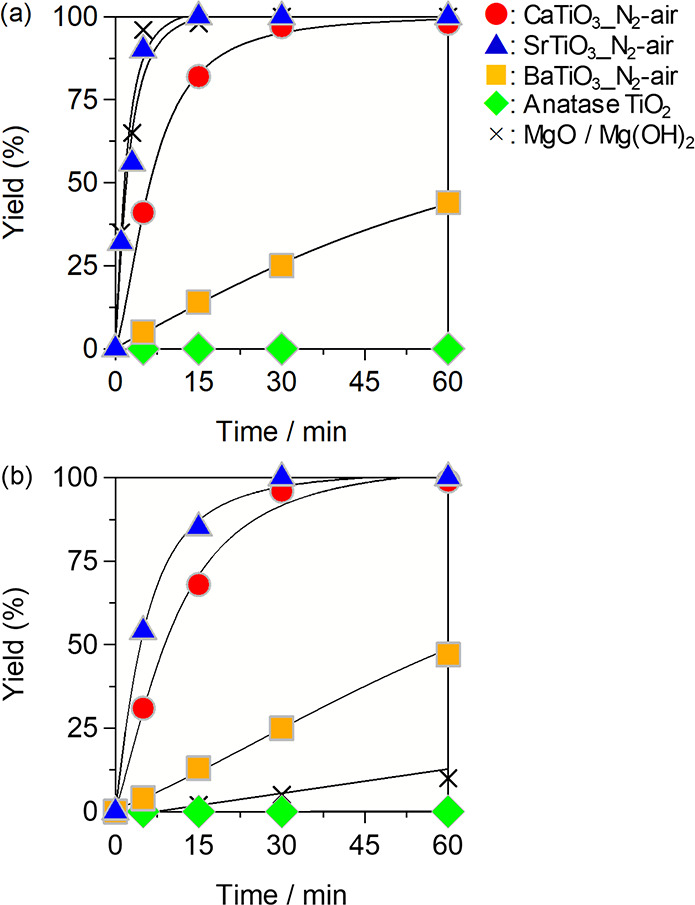
Cyanosilylation of **1a** with TMSCN over *A*TiO_3__N_2_-air (a) pretreated at 573 K for 1 h *in vacuo* and (b) without pretreatment. Conditions: catalyst (50 mg), **1a** (1.0 mmol), TMSCN (1.5 mmol), toluene (2 mL), ice bath (275 K), Ar atmosphere. GC yields are given. Mg(OH)_2_ (72.3 mg; i.e., 50 mg as MgO) was used.

First, we compared the catalytic activity of *A*TiO_3__N_2_-air with those of anatase TiO_2_ and MgO prepared *in situ* from Mg(OH)_2_ as representative heterogeneous acid and base catalysts, respectively, with pretreatment at 573 K for 1 h *in vacuo*. Figure 5a shows the time course of the cyanosilylation of **1a** with TMSCN over the catalysts. The reaction quantitatively proceeded to give **2a** over MgO, SrTiO_3__N_2_-air, and CaTiO_3__N_2_-air, whereas the yields of **2a** with BaTiO_3__N_2_-air and MgTiO_3__N_2_-air were moderate, and anatase TiO_2_ was inactive. The reaction rate decreased in the order MgO (6.9 mmol g^–1^ min^–1^) ∼ SrTiO_3__N_2_-air (6.3 mmol g^–1^ min^–1^) > CaTiO_3__N_2_-air (1.7 mmol g^–1^ min^–1^) > BaTiO_3__N_2_-air (2.0 × 10^–1^ mmol g^–1^ min^–1^) > > anatase TiO_2_. Remarkably, the present perovskite oxides could function as effective heterogeneous catalysts without pretreatment of the catalysts before the reaction. The reaction profiles over the catalysts without a pretreatment are shown in Figure 5b. Although the catalytic activity of Mg(OH)_2_ decreased substantially, in sharp contrast to MgO (pretreated Mg(OH)_2_), the reactions over SrTiO_3__N_2_-air and CaTiO_3__N_2_-air smoothly proceeded even without a pretreatment. In addition, the present SrTiO_3__N_2_-air-catalyzed system was applicable to a larger-scale cyanosilylation of **1a** with TMSCN at 298 K, which resulted in the isolation of analytically pure **2a** (2.06 g) (eq 1). In this case, the formation rate for **2a** over SrTiO_3__N_2_-air at 298 K reached 8.4 mmol g^–1^ min^–1^ even without a pretreatment, and this value was the highest among those reported for solid-catalyst-mediated systems that did not require thermal pretreatments (6.9 × 10^–3^ to 3.9 mmol g^–1^ min^–1^; see Table S2). In addition, the reaction efficiently proceeded under solvent-free conditions, with a **2a** formation rate of 2.1 × 10^1^ mmol g^–1^ min^–1^ (entry 2, Table 2),^63^ which is comparable to the formation rates achieved with the most active pretreated catalysts, such as Sn-Mont^62^ (9.8 × 10^1^ mmol g^–1^ min^–1^) and Al-MCM-41^61^ (3.6 × 10^1^ mmol g^–1^ min^–1^).
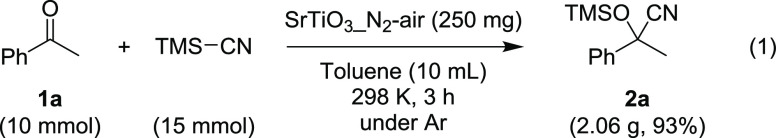
1

**Table 2:**
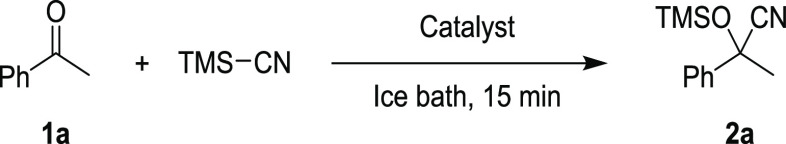
Cyanosilylation of Acetophenone (**1a**) with TMSCN over Various Catalysts^a^

entry	catalyst	yield (%)	entry	catalyst	yield (%)
1	CaTiO_3__N_2_-air	68	19	Nb_2_O_5_	<1
2	SrTiO_3__N_2_-air	85 (99)^b^	20	CeO_2_	3
3	BaTiO_3__N_2_-air	13	21	Na_2_CO_3_	<1
4	CaTiO_3__air	34	22	SrCO_3_	<1
5	SrTiO_3__air	23	23	MgTiO_3__N_2_-air	9
6	BaTiO_3__air	6	24	SiO_2_-MgO	<1
7	CaTiO_3__purchased	<1	25	H-ZSM-5 (90)^d^	<1
8	SrTiO_3__purchased	<1	26	H-β (390)^d^	<1
9	BaTiO_3__purchased	<1	27	H-Y (4.55)^d^	<1
10	Mg(OH)_2_^c^	2	28	Mont. K-10	74
11	γ-Al_2_O_3_	2	29	hydrotalcite	71
12	SiO_2_	<1	30	hydroxyapatite	<1
13	anatase TiO_2_	<1	31	SO_4_^2–^/ZrO_2_	<1
14	rutile TiO_2_	<1	32	Amberlyst 15	24
15	β-MnO_2_	<1	33	Nafion NR-50	<1
16	α-Fe_2_O_3_	<1	34	*p*-TsOH^e^	<1
17	ZnO	<1	35	pyridine^e^	<1
18	ZrO_2_	<1	36	blank	<1

a Reaction conditions: catalyst (50 mg), **1a** (1.0 mmol), TMSCN (1.5 mmol), toluene (2 mL), ice bath (275 K), 15 min, Ar atmosphere. Catalysts were not treated before use in a catalytic run. The yield was determined by GC.

b Solvent-free conditions: catalyst (50 mg), **1a** (1.0 mmol), TMSCN (1.5 mmol), 298 K, 1 min under Ar.

c 72.3 mg of Mg(OH)_2_ (as 50 mg of MgO) was used.

d The SiO_2_/Al_2_O_3_ ratio of zeolites.

e Homogeneous catalyst (0.1 mol L^–1^).

Next, the catalytic performance of SrTiO_3__N_2_-air was compared with that of various solid catalysts, including simple and crystalline complex oxides not subjected to a thermal pretreatment (Table 2). As mentioned, *A*TiO_3__air was substantially more active than *A*ZrO_3__air and *A*NbO_3__air (Figure 2c). The **2a** yield achieved with *A*TiO_3__N_2_-air was greater than that achieved with *A*TiO_3__air, and commercially available titanates with low specific surface areas (1–4 m^2^ g^–1^) were inactive, indicating the effectiveness of the present synthesis procedure of changing the calcination atmosphere from N_2_ to air. Other metal oxides (γ-Al_2_O_3_, SiO_2_, anatase TiO_2_, rutile TiO_2_, β-MnO_2_, α-Fe_2_O_3_, ZnO, ZrO_2_, Nb_2_O_5_, and CeO_2_), carbonates (Na_2_CO_3_ and SrCO_3_), zeolites (H-ZSM-5, H-β, and H-Y), homogeneous catalysts (*p*-toluenesulfonic acid (TsOH) and pyridine), and acid–base materials (Mg(OH)_2_, SiO_2_–MgO, SO_4_^2–^/ZrO_2_, hydroxyapatite (Ca_10_(PO_4_)_6_(OH)_2_), and Nafion NR-50) were almost inactive toward the cyanosilylation of **2a** under the present reaction conditions.^1,2^ In addition, MgTiO_3__N_2_-air with an ilmenite-type structure and Amberlyst 15 produced **2a** in low yields. Hydrotalcite (Mg_6_Al_2_(OH)_16_CO_3_·4H_2_O), which has been reported to be active for cyanosilylation,^63^ and Montmorillonite K-10 (Mont. K-10) gave **2a** in 71 and 74% yields, respectively; however, further reaction did not proceed over either catalyst when the reaction time was prolonged. The reaction also efficiently proceeded over SrTiO_3__N_2_-air with an equivalent amount of TMSCN (76% at 15 min, 84% at 90 min); however, the **2a** yield was lower than that attained with excess TMSCN, likely because of the decomposition of TMSCN.

**Figure 6:**
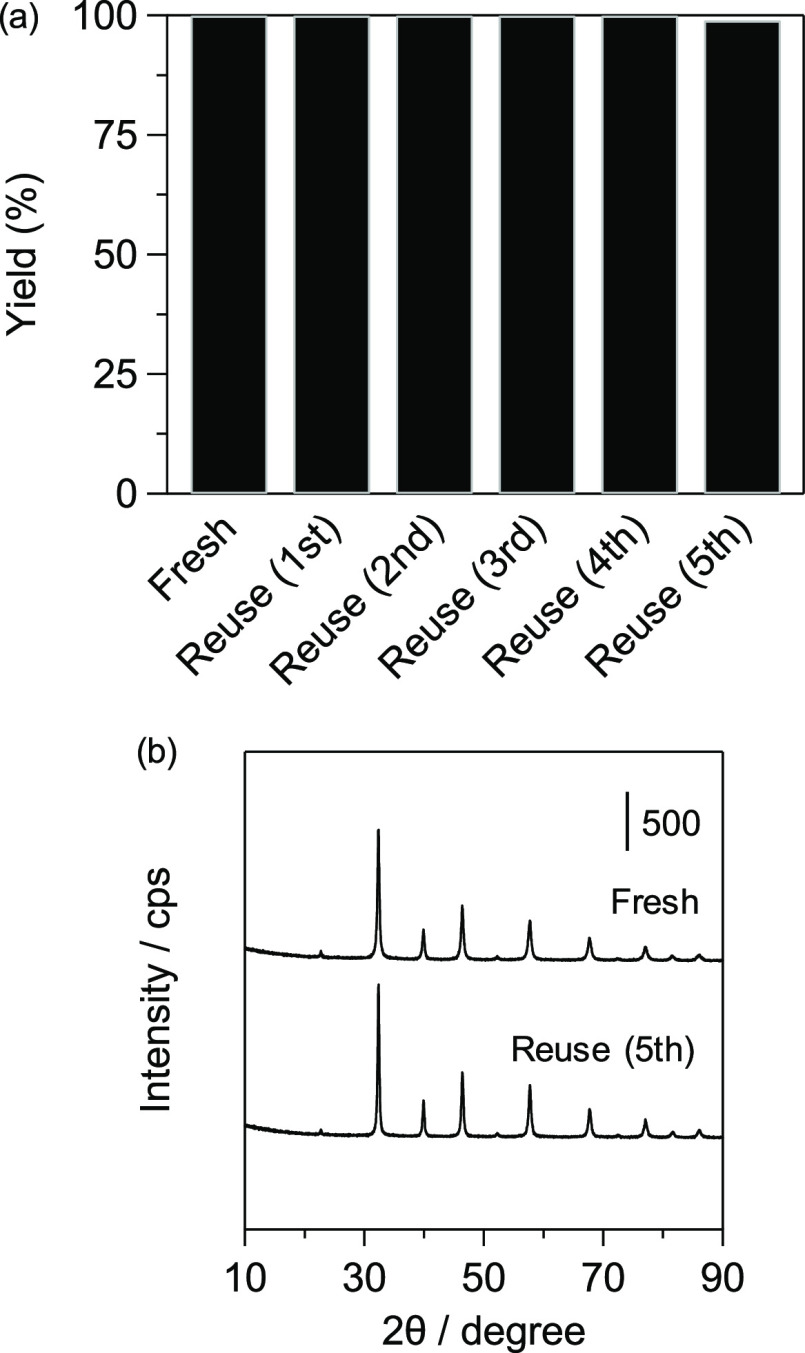
(a) Reusability of SrTiO_3__N_2_-air for cyanosilylation of **1a** with TMSCN. Conditions: SrTiO_3__N_2_-air (50 mg), **1a** (1.0 mmol), TMSCN (1.5 mmol), toluene (2 mL), ice bath (275 K), 30 min, Ar atmosphere. Yields were determined by GC. (b) XRD patterns for fresh SrTiO_3__N_2_-air and SrTiO_3__N_2_-air catalysts after they were reused five times.

To confirm the heterogeneous nature of SrTiO_3__N_2_-air, we investigated its reusability and stability during catalysis. After the cyanosilylation of **1a** with TMSCN was carried out under the conditions described in Figure 5b, the used SrTiO_3__N_2_-air catalyst could be easily recovered from the reaction mixture by simple filtration. The recovered catalyst was reused five times without a substantial loss of catalytic performance (Figure 6a). ICP–AES analysis showed negligible leaching of Sr or Ti species into the filtrate (both <0.01% with respect to fresh SrTiO_3__N_2_-air); therefore, the present cyanosilylation of **1a** with TMSCN proceeds on the solid surface of SrTiO_3__N_2_-air. In addition, no significant difference was observed between the XRD patterns for the fresh and used catalyst (Figure 6b), suggesting that SrTiO_3__N_2_-air is stable during the catalytic reaction.

The SrTiO_3__N_2_-air catalyst was also applicable to the cyanosilylation of various carbonyl compounds with TMSCN (Table 3). Various aromatic and aliphatic ketones were efficiently converted into the corresponding cyanohydrin trimethylsilyl ethers in high-to-excellent yields over SrTiO_3__N_2_-air without heat treatment before the reaction. The reaction of **1a** as well as acetophenones with electron-donating and electron-withdrawing *para* substituents (**1d**–**1i**) proceeded to form the corresponding products in excellent yields (**2d**–**2i**). Although the reactivity of *m*-methyl acetophenone (**1c**) was comparable to that of **1a**, the reaction rate for *o*-methyl acetophenone (**1b**) was lower than those for **1a** and **1c**, likely because of steric hindrance between the active sites and methyl group at the *ortho* position. The reaction of cyclopropyl phenyl ketone (**1j**) proceeded selectively without opening of the cyclopropyl ring. In addition to monoaryl ketones, SrTiO_3__N_2_-air efficiently catalyzed the cyanosilylation of diaryl ketones (benzophenone (**1k**) and 9-fluorenone (**1l**)) and cyclic and acyclic aliphatic ketones (cyclohexanone (**1m**), 2-adamantanone (**1n**), 2-octanenone (**1o**), and 4-methyl-2-pentanone (**1p**)). Moreover, the reaction of benzaldehyde (**1q**) proceeded more easily to give the corresponding product (**2q**) even when a small amount of catalyst was used. An α,β-unsaturated carbonyl compound such as *trans*-cinnamaldehyde (**1r**) was smoothly converted into the corresponding 1,2-adduct as the sole product. The reaction of 2-cyclohexen-1-one (**1s**) mainly gave the 1,2-adduct (**2s**) along with a small amount of bis-adduct (**2s′**), which was produced via the extra cyanosilylation of 3-oxocyclohexane-1-carbonitrile formed through the hydrolysis of the 1,4-adduct, in a ratio of 98/2. Onaka and co-workers reported that typical solid bases (MgO, CaO, and hydroxyapatite) and strong solid acids (Al-, Fe-, and Sn-Mont) predominately gave 1,2- and 1,4-adducts, respectively;^60,64^ thus, the reaction over the SrTiO_3__N_2_-air catalyst might proceed mainly on base sites.

**Table 3:**
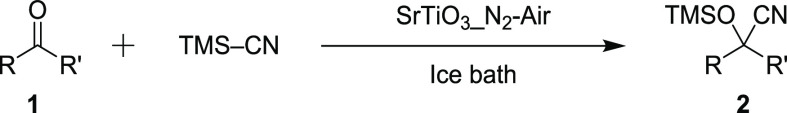

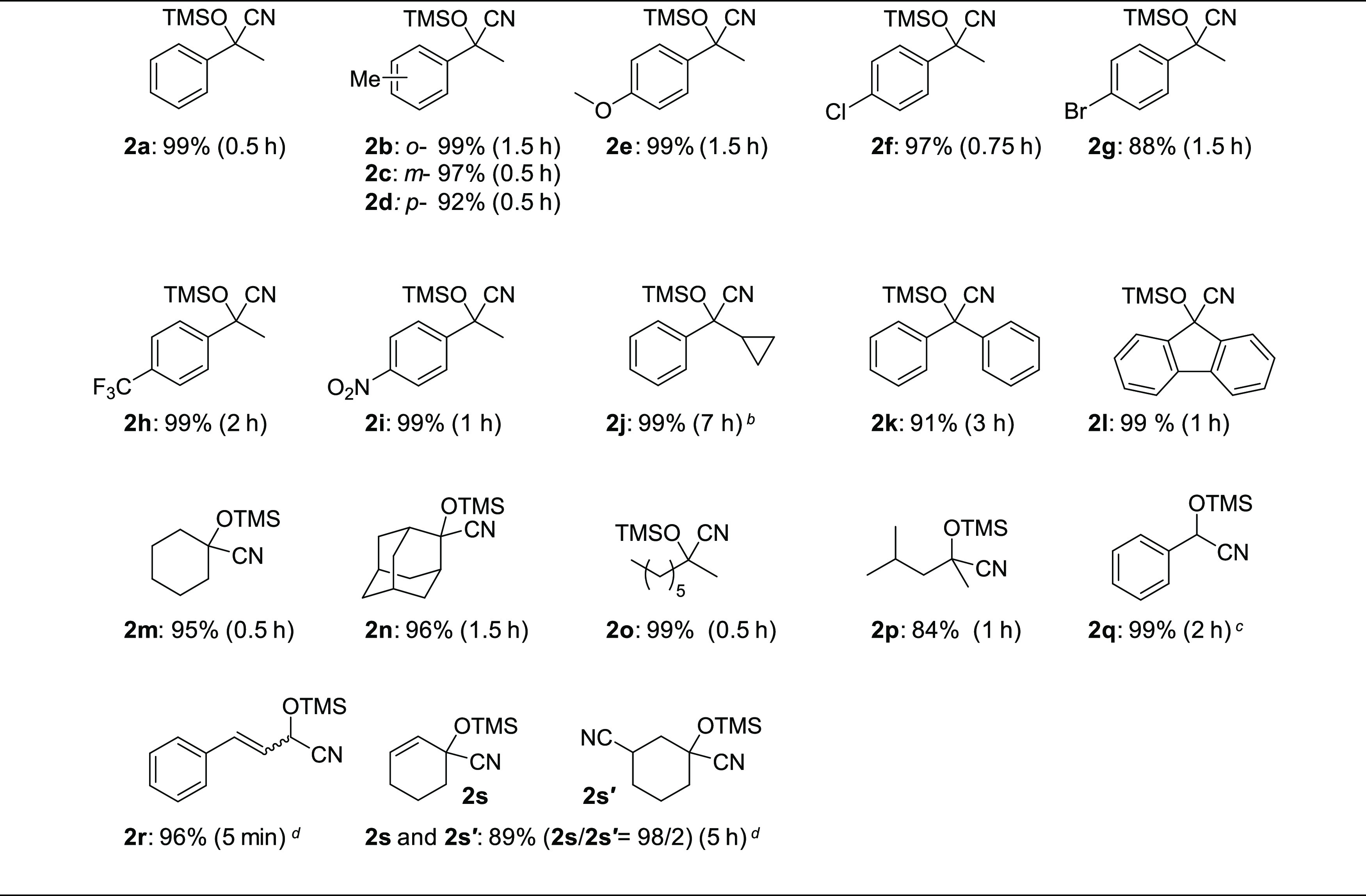
Scope of Substrate for Cyanosilylation of Various Carbonyl Compounds with TMSCN over SrTiO_3__N_2_-air Catalyst^a^

a Reaction conditions: SrTiO_3__N_2_-air (50 mg), **1** (1.0 mmol), TMSCN (1.5 mmol), toluene (2 mL), ice bath (275 K), Ar atmosphere. Isolated yields were given.

b TMSCN (3.0 mmol).

c SrTiO_3__N_2_-air (10 mg).

d GC yield.

### 3.3 Acid–Base Properties of Various Perovskite Oxides

**Figure 7:**
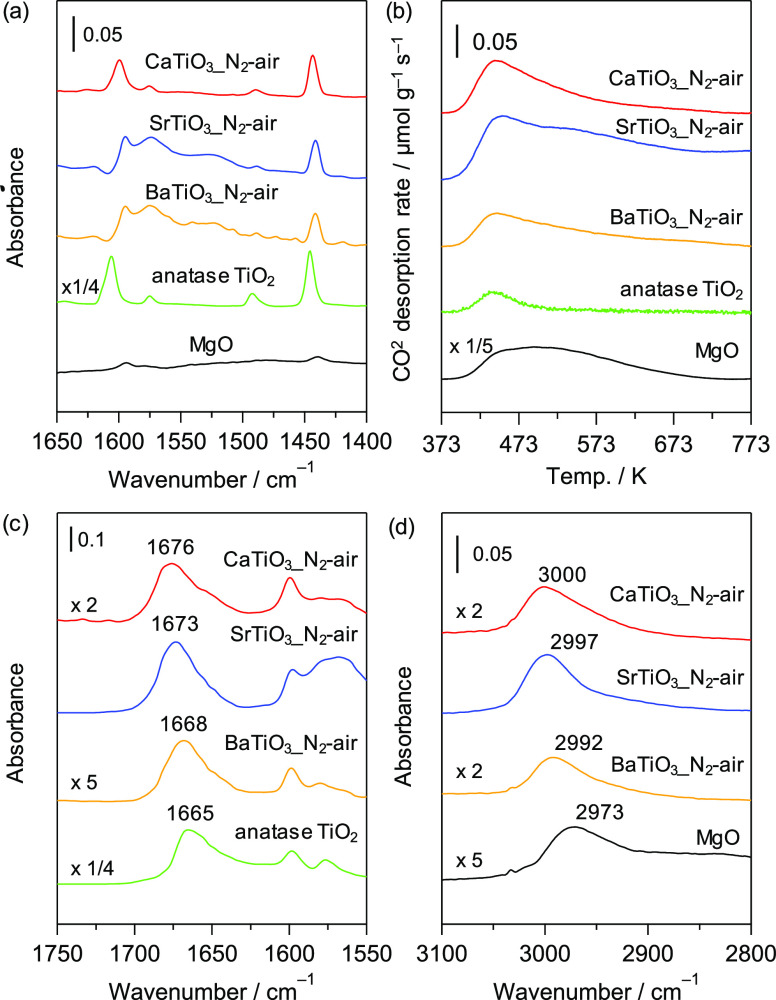
Difference FT-IR spectra of (a) pyridine, (d) CHCl_3_ in νC–H region, (c) acetophenone adsorbed onto Ti-based perovskite oxides; (b) CO_2_-TPD profiles of *A*TiO_3__N_2_-air.

The synthesized Ti-based perovskite oxides (in particular, SrTiO_3__N_2_-air) strongly promoted the cyanosilylation of **1a** with TMSCN, in sharp contrast to various other metal oxides, including well-known solid acid and/or base catalysts. To investigate the high catalytic activity of SrTiO_3__N_2_-air, we characterized its acid and base properties via FT-IR and TPD measurements using probe molecules such as pyridine, CO_2_, acetophenone, and chloroform; the results are summarized in Table 4. First, the amounts of acid and base sites on various catalysts were investigated by FT-IR spectroscopy for a sample with absorbed pyridine and by CO_2_-TPD measurements, respectively. Figure 7a shows difference IR spectra of pyridine adsorbed onto the catalysts (*A*TiO_3__N_2_-air, anatase TiO_2_, and MgO). In all cases, bands at 1450, 1500, 1575, 1600, and 1630 cm^–1^, which are ascribed to the 19b, 19a, 8b, 8a, and 1+6a vibration modes of pyridine coordinated to a Lewis acid site, respectively, were observed.^65^ Notably, the band at 1550 cm^–1^, which corresponds to pyridinium ions, was not observed in any of the spectra, indicating that all the catalysts lack Brønsted acid sites. The amount of Lewis acid sites was estimated from the area of the band at 1450 cm^–1^ and its integrated molar extinction coefficient (2.22 cm μmol^–1^).^66^ Although MgO had almost no Lewis acid sites, the synthesized titanates and anatase TiO_2_ showed moderate-to-large amounts of Lewis acid sites (Table 4). The amount of Lewis acid sites decreased in the order of anatase TiO_2_ (132 μmol g^–1^) > > CaTiO_3__N_2_-air (27 μmol g^–1^) > BaTiO_3__N_2_-air (20 μmol g^–1^) ∼ SrTiO_3__N_2_-air (19 μmol g^–1^) > MgO (4 μmol g^–1^).

**Table 4:** Acid–Base Properties of Ti-Based Perovskite Oxides

entry	catalyst	rate^a^/mmol g^–1^ min^–1^	Lewis acidity^b^/μmol g^–1^	*ν*C=O^c^/cm^–1^	basicity^d^/μmol g^–1^	νC–H^e^/cm^–1^
1	CaTiO_3__N_2_-air	1.7	27	1676	60	3000
2	SrTiO_3__N_2_-air	6.3	19	1673	137	2997
3	BaTiO_3__N_2_-air	2.0 × 10^–1^	20	1668	45	2992
4	anatase TiO_2_		132	1665	13	f
5	MgO	6.9	4	f	440	2973

a The cyanosilylation rate of **1a** with TMSCN over the catalysts pretreated at 573 K for 1 h *in vacuo*.

b Estimated from the band at 1450 cm^–1^ of adsorbed pyridine (ε = 2.22 cm μmol^–1^).^66^

c The band position of νC=O of adsorbed acetophenone.

d Estimated from CO_2_-TPD measurement.

e The position of *ν*C–H in adsorbed CHCl_3_ species.

f Not measured.

We next used CO_2_-TPD measurements to estimate the basic properties of titanates (Figure 7b). Although desorption peaks at ∼450 K were observed for titanates and anatase TiO_2_, MgO showed much larger and broader desorption peaks than the Ti-based materials, which suggests a high content of strong base sites on MgO. The amount of base sites estimated from the amount of desorbed CO_2_ decreased in the order of MgO (440 μmol g^–1^) > > SrTiO_3__N_2_-air (137 μmol g^–1^) > CaTiO_3__N_2_-air (60 μmol g^–1^) > BaTiO_3__N_2_-air (45 μmol g^–1^) > anatase TiO_2_ (13 μmol g^–1^). No relationship was observed between the amount of acid/base sites and catalytic activity with pretreatment (SrTiO_3__N_2_-air ∼ MgO > CaTiO_3__N_2_-air > BaTiO_3__N_2_-air > > anatase TiO_2_); thus, the high catalytic activity of SrTiO_3__N_2_-air cannot be simply explained by these parameters. Although the amount of acid sites for SrTiO_3__air was approximately the same as that for SrTiO_3__N_2_-air, as evaluated from FT-IR measurements of adsorbed pyridine, the CO_2_-TPD measurements revealed that the amount of base sites for SrTiO_3__N_2_-air was 2.5 times greater than that for SrTiO_3__air (52 μmol g^–1^). The surface structure of SrTiO_3_ was analyzed by XPS (Figure S7). No substantial difference in peak positions corresponding to Ti 2*p*, Sr 3*p*, and O 1*s* was observed between SrTiO_3__N_2_-air and SrTiO_3__air; however, the surface atomic ratio (Sr/Ti) for SrTiO_3__N_2_-air was estimated to be 1.51, which is higher than that for SrTiO_3__air (1.37), suggesting that the enrichment of Sr–O termination at the surface of SrTiO_3__N_2_-air leads to an increase in the amount of base sites. Wu and co-workers also reported that enrichment of alkaline-earth metals or transition metals at the surface of perovskite oxides is important for achieving acid–base properties characteristic of their surfaces and can be controlled by thermal and chemical treatments;^37^ thus, N_2_ treatment during the calcination step increased the amount of base sites over SrTiO_3__N_2_-air and the cyanosilylation activity.

**Figure 8:**
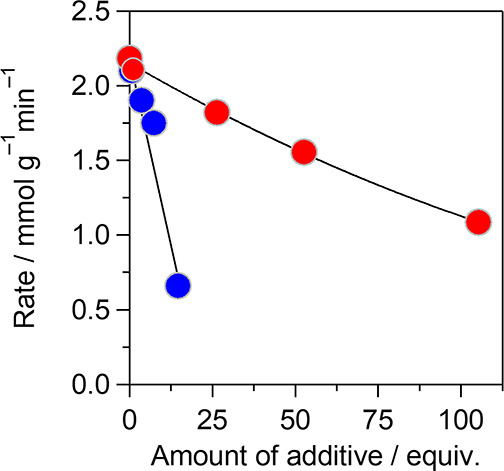
Poisoning effect of pyridine (red circle) and AcOH (blue circle) for cyanosilylation of **1a** with TMSCN over SrTiO_3__N_2_-air catalyst. Conditions: SrTiO_3__N_2_-air (50 mg), **1a** (1.0 mmol), TMSCN (1.5 mmol), toluene (2 mL), additive (pyridine and AcOH: approximately 1–100 and 1–25 equivalents with respect to acid and base sites of SrTiO_3__N_2_-air, respectively), ice bath (275 K), Ar atmosphere.

Because SrTiO_3__N_2_-air has a large amount of base sites and a similar amount of Lewis acid sites compared with other titanates, the simultaneous presence of acid and base sites likely plays an important role in the present cyanosilylation. To investigate the possible cooperative acid–base catalysis of SrTiO_3__N_2_-air for cyanosilylation of carbonyl compounds, we carried out the reactions by adding basic and acidic molecules (pyridine (approximately 1–100 equivalents with respect to the acid sites of SrTiO_3__N_2_-air) and AcOH (approximately 1–25 equivalents with respect to the base sites of the catalyst)) to poison the acidic and basic active sites on SrTiO_3__N_2_-air, respectively (Figure 8). The reaction rate decreased with increasing amounts of both pyridine and AcOH, suggesting that both the acid and base sites contribute to the cyanosilylation. Moreover, the poisoning effect with AcOH was much stronger than that with pyridine. These results suggest that the reaction is mainly promoted by the base sites with possible cooperative action of the Lewis acid sites on the SrTiO_3__N_2_-air catalyst. This supposition is supported by the two following results: (i) MgO with a large amount of strong base sites was highly active for cyanosilylation. (ii) Despite a smaller amount of base sites on SrTiO_3__N_2_-air than that on MgO, the catalytic activities of SrTiO_3__N_2_-air and MgO were similar. Among perovskites containing *d*^0^-transition metals investigated in the present study, the series of Ti-based perovskite oxides showed the highest catalytic performance for the cyanosilylation of **1a** with TMSCN. Although the *A*TiO_3_ catalysts had both acid and base sites on their surface, the Zr- and Nb-based perovskite oxides showed only basicity and almost no acidity, as estimated by TPD measurements (Figure S8). These results also indicate that the coexistence of acid and base sites on perovskite oxides plays an important role in their high catalytic activity.

**Figure 9:**
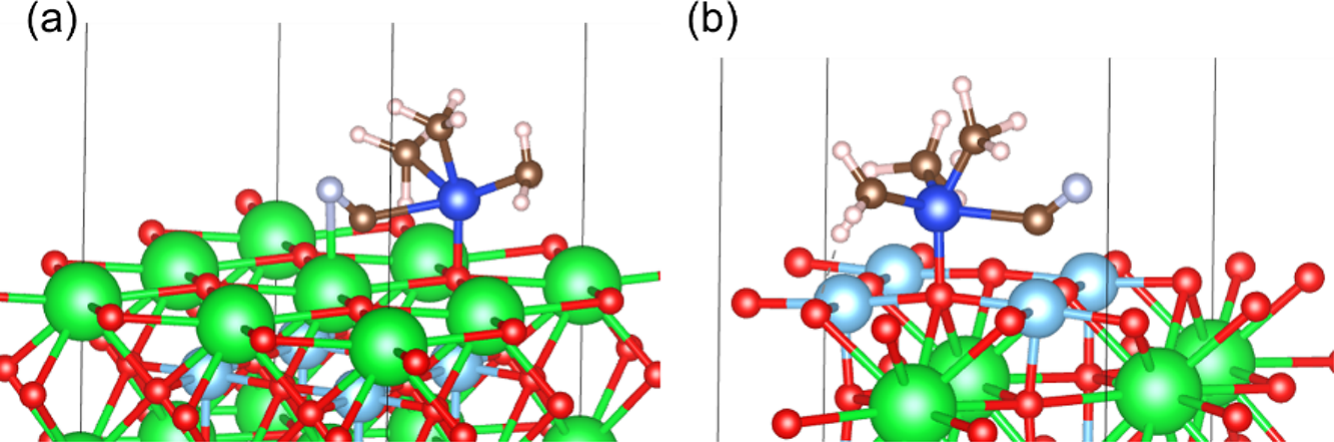
Adsorption configurations of TMSCN on SrTiO_3_ for (a) Sr-terminated (100) and (b) Ti-terminated (100) surfaces from DFT geometry optimization. Green, light blue, red, blue, brown, light purple, and light red spheres represent Sr, Ti, O, Si, C, N, and H atoms, respectively.

**Scheme 1:**
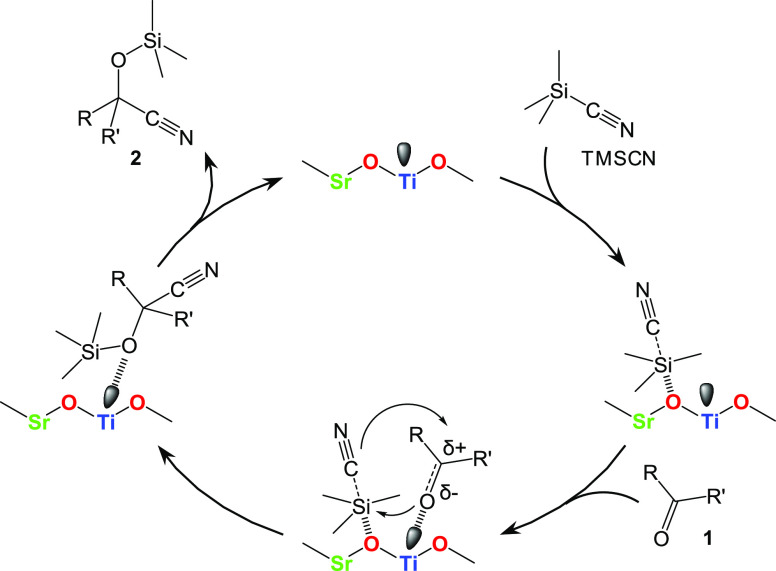
Possible Reaction Mechanism for Cyanosilylation of Carbonyl Compounds with TMSCN over SrTiO_3__N_2_-air Catalyst

Scheme 1 shows a possible cyanosilylation mechanism over the SrTiO_3__N_2_-air catalyst; this mechanism is based on the results of our investigation of acid and base properties. First, TMSCN is activated by surface oxygen acting as a base site. Similarly, carbonyl compounds (**1**) are adsorbed and activated by Lewis acid sites on the catalyst surface. Such cooperative activation facilitates a nucleophilic attack of a CN^–^ anion at the carbon atom of the carbonyl groups, followed by desorption of the corresponding cyanohydrin trimethylsilyl ether (**2**). On the basis of the chemoselectivity of **1s** and the poisoning effect for cyanosilylation over SrTiO_3__N_2_-air, the reaction would be mainly promoted by base sites, and the activation of TMSCN by base sites on SrTiO_3_ is likely a key step. Therefore, the DFT calculations were conducted to estimate the interaction of TMSCN with SrTiO_3_. The pristine facet (100) can be fully terminated on either Sr or Ti,^31^ and two model surfaces of Sr-terminated and Ti-terminated (100) facets were used. The adsorption energy of chemisorbed TMSCN on the Sr-terminated (100) was calculated to be −2.01 eV and lower than that (−1.01 eV) on the Ti-terminated (100) facet (Figure 9), which suggests that TMSCN is strongly activated on the SrO-rich surface. These results are consistent with the higher reactivity of SrTiO_3__N_2_-air with large amounts of base sites than that of SrTiO_3__air.

The effect of the acid/base strength of catalysts on cyanosilylation was investigated by FT-IR measurements with adsorbed **1a** and CHCl_3_. The FT-IR spectrum of **1a** adsorbed onto SrTiO_3__N_2_-air revealed that the νC=O band of **1a** clearly shifted to a lower wavenumber (1673 cm^–1^) compared with its position in the spectrum of a gas-phase sample of **1a** (1710 cm^–1^) (Figure 7c), supporting the activation of carbonyl groups on Lewis acid sites of SrTiO_3__N_2_-air. The order of the magnitude of the νC=O band shift was anatase TiO_2_ (1665 cm^–1^) > BaTiO_3_ (1668 cm^–1^) > SrTiO_3_ (1673 cm^–1^) > CaTiO_3_ (1676 cm^–1^).

The basic strength of the catalysts was further estimated by FT-IR analysis of CHCl_3_. Figure 7d shows the *ν*C–H region of the FT-IR spectra of adsorbed CHCl_3_. The acidic hydrogen of the CHCl_3_ molecule can interact with base sites on a solid surface, and the strength of base sites can be estimated from the magnitude of the shift of the *ν*C–H band for CHCl_3_ from its original position (3019 cm^–1^ for gas-phase CHCl_3_).^67^ The bands corresponding to *ν*C–H for adsorbed CHCl_3_ were clearly shifted to lower wavenumbers, and their red-shift order was MgO (2973 cm^–1^) > > BaTiO_3__N_2_-air (2992 cm^–1^) > SrTiO_3__N_2_-air (2997 cm^–1^) > CaTiO_3__N_2_-air (3000 cm^–1^). All of these results suggest a moderate amount and moderate strength of acid and base sites on the SrTiO_3__N_2_-air catalyst compared with those on MgO and anatase TiO_2_. These results also suggest that such acid and base sites can function as active sites for cyanosilylation without a heat pretreatment of the catalyst.

The differences in the crystal structures of *A*TiO_3_ (orthorhombic CaTiO_3_, cubic SrTiO_3_, and tetragonal BaTiO_3_) possibly affect their acid–base properties and surface structures because of distortion of the octahedral TiO_6_ units. Therefore, we investigated the *A*-site effect on the intrinsic reactivity of each *A*TiO_3__N_2_-air sample. Although the most active sample, SrTiO_3__N_2_-air, exhibited the highest density of base sites among the investigated titanates, a good linear relationship between the acid and base densities and the reaction rate per surface area was not confirmed (Figure S9). This discrepancy cannot be explained even when considering the order of the strength of acid and base sites estimated from the FT-IR results (BaTiO_3__N_2_-air > SrTiO_3__N_2_-air > CaTiO_3__N_2_-air). The distance between acid and base sites might also be important for cooperative acid–base catalysis. Figure S10 shows the δH–C–Cl region of the FT-IR spectra of adsorbed CHCl_3_, where sharp bands at ∼1220 cm^–1^, which are attributed to CHCl_3_ adsorbed onto base sites, are observed in the spectra of all of the titanates.^67^ By contrast, a shoulder band at ∼1240 cm^–1^, which is commonly assigned to a bidentate CHCl_3_ species interacting between an acidic hydrogen–basic site and basic chlorine–acidic site, clearly appeared in the spectrum of SrTiO_3__N_2_-air but not in the spectra of the Ca- or Ba-based samples, suggesting the presence of neighboring acid–base pair sites. Acid–base bifunctional catalysts have been reported to show high catalytic performance for the cyanosilylation of carbonyl compounds with TMSCN because of the activation of both substrates.^13,20^ The highly dense base sites and nearby Lewis acid sites likely caused effective activation of **1** and TMSCN, promoting the cyanosilylation reaction and resulting in the high catalytic activity of SrTiO_3__N_2_-air.

## 4 Conclusions

Nanosized Ti-, Zr-, and Nb-based perovskite oxide particles with high purity were successfully synthesized by a simple sol–gel method using dicarboxylic acid without the need for specific reagents, a multistep procedure, or post-treatment. In particular, the specific surface area of Ti-based perovskite oxides could be increased by changing the calcination atmosphere from N_2_ to air. SrTiO_3__N_2_-air with a high specific surface area (46 m^2^ g^–1^) could act as an effective and reusable solid catalyst for cyanosilylation of various types of aromatic and aliphatic carbonyl compounds with TMSCN under mild conditions and without a thermal pretreatment. Detailed surface analysis using FT-IR and TPD measurements of various probe molecules, along with poisoning tests, revealed that both a moderate amount and moderate strength of acid and base sites on the SrTiO_3_ catalyst play an important role in cooperatively activating carbonyl compounds and TMSCN without a heat pretreatment, in sharp contrast to acidic anatase TiO_2_ and basic MgO catalysts. The results of this study suggest that nanostructured perovskite oxides are effective bifunctional solid catalysts with controllable acid and base sites. This approach of high functionalization of perovskite oxides with versatile compositions and structures is a promising strategy for developing highly efficient liquid-phase organic reactions that proceed under mild conditions.

## Supporting Information Available

Experimental details, 2 tables (comparison of previously reported SrTiO_3_ and cyanosilylation systems), and 10 figures (XRD patterns, TG–DTA profiles, XAFS spectra, SEM images, TEM images, XP spectra, NH_3_- and CO_2_-TPD profiles, and difference IR spectra of CHCl_3_ and NMR spectra) (PDF)
