# Supplementary material for: Nanosized Ti-Based Perovskite Oxides as Acid–Base Bifunctional Catalysts for Cyanosilylation of Carbonyl Compounds
Source: ACS Appl Mater Interfaces. 2023 Apr 3;15(14):17957–68. doi: 10.1021/acsami.3c01629 (PMC10103063; doi:10.1021/acsami.3c01629)
Supplement: Supplementary file 1 — am3c01629_si_001.pdf [file am3c01629_si_001.pdf]

Supporting information

Nanosized Ti-based Perovskite Oxides as Acid–Base Bifunctional Catalysts for Cyanosilylation of Carbonyl Compounds

Takeshi Aihara[†], *Wataru Aoki*[†], *Shin Kiyohara*[‡], *Yu Kumagai*[‡], *Keigo Kamata*[*,†], and *Michikazu Hara*[*,†]

[†] Laboratory for Materials and Structures, Institute of Innovative Research, Tokyo Institute of Technology, Nagatsuta-cho 4259, Midori-ku, Yokohama 226-8503, Japan

[‡] Institute for Materials Research, Tohoku University, 2-1-1 Katahira, Aoba-ku, Sendai 980-8577, Japan

Email address: kamata.k.ac@m.titech.ac.jp

Experimental Details

Materials. Magnesium acetate tetrahydrate ($Mg(OAc)_2·4H_2O$), calcium acetate monohydrate ($Ca(OAc)_2·H_2O$), strontium acetate hydrate ($Sr(OAc)_2·1/2H_2O$), barium acetate ($Ba(OAc)_2$), titanium(IV) isopropoxide ($Ti(O\textit{i}\text{-}Pr)_4$), DL-malic acid, aspartic acid, citric acid, *n*-decane, acetic acid (AcOH), *p*-toluenesulfonic acid monohydrate (*p*-TsOH), pyridine, and benzophenone were

purchased from Kanto Chemical (Japan). Zirconium oxyacetate ($ZrO(OAc)_2$) aqueous solution, acetophenone, 2′-methylacetophenone, 3′-methylacetophenone, 4′-methylacetophenone, 4′-methoxyacetophenone, 4′-chloroacetophenone, 4′-bromoacetophenone, 4′-nitroacetophenone, cyclopropyl phenyl ketone, 9-fluorenone, 2-adamantanone, 2-octanone, 4-methyl-2-pentanone, *trans*-cinnamaldehyde, and TMSCN were obtained from Tokyo Chemical Industry (Japan). H_2O_2 (30% aqueous solution), cyclohexanone, benzaldehyde, and chloroform were obtained from FUJIFILM Wako Pure Chemical (Japan). 4′-Trifluoromethylacetophenone was obtained from Sigma-Aldrich. Niobium(V) ethoxide ($Nb(OEt)_5$) was purchased from Kojundo Chemical Laboratory. Ti-based perovskite oxides such as $CaTiO_3$, $SrTiO_3$, and $BaTiO_3$ were obtained from Kojundo Chemical Laboratory. TiO_2_anatase (ST-01) and TiO_2_rutile were obtained from Ishihara Sangyo Kaisha (Japan) and Wako Pure Chemical (Japan), respectively. Hydroxyapatite ($Ca_{10}(PO_4)_6(OH)_2$, monoclinic), and hydrotalcite ($Mg_6Al_2(OH)_{16}CO_3 \cdot 4H_2O$) were obtained from FUJIFILM Wako Pure Chemical (Japan). $Mg(OH)_2$, β-MnO_2, α-Fe_2O_3, ZnO, Amberlyst 15 (hydrogen form DRY), Nafion NR-50, and Montmorillonite K-10 (Mont. K-10) were purchased from Sigma-Aldrich. γ-Al_2O_3 (JRC-ALO-9), ZrO_2 (JRC-ZRO-6), Nb_2O_5 (JRC-NBO-2), CeO_2 (JRC-CEO-3), H-ZSM-5 (JRC-Z5-90H, SiO_2/Al_2O_3 = 90), SO_4^{2-}/ZrO_2 (JRC-SZ-1), and SiO_2–MgO (JRC-SM-2) were supplied by the Catalysis Society of Japan. SiO_2 (CARiACT Q-10) was obtained from FUJI SILYSIA CHEMICAL (Japan). Na_2CO_3 and $SrCO_3$ were purchased from Kanto Chemical (Japan). H-β (SiO_2/Al_2O_3 = 390), and H-Y (SiO_2/Al_2O_3 = 4.55) were purchased from Alfa Aesar (U.K.). Toluene was pretreated with molecular sieves (3A) evacuated at 553 K for 3 h.[S1] TMSCN was distilled before being used in a catalytic run.[S2]

Catalyst preparation. Titanates were synthesized by a sol–gel method. First, 20 mmol of dl-malic acid and 20 mmol of H_2O_2 were dissolved in H_2O (200 mL), followed by the dropwise addition of 5 mmol of Ti(O*i*-Pr)$_4$; the resultant mixture was stirred until the added Ti(O*i*-Pr)$_4$ was completely dissolved. *A*-site metal acetates (5 mmol) were then added, and the solvent was evaporated. The resultant red–orange solid was dried at 463 K for 1 h to give a pale-yellow powder referred to here as the precursor. The obtained precursor was treated using two methods to obtain titanates: (1) treatment under N_2 in a process where the temperature was gradually increased (ramp: 3 K min^{-1}) and the atmosphere was subsequently changed to air for calcination at the desired temperature for 5 h; and (2) calcination under air at the desired temperature for 5 h. $MgTiO_3$ with an ilmenite-type structure was also obtained using the same procedure for the other titanates, followed by calcination at 873 K for 5 h. Zirconates were synthesized via the same procedure using $Zr(OAc)_2$, alkaline-earth-metal acetates, and aspartic acid as a dicarboxylic acid. Niobates were prepared via the same procedure using $Nb(OEt)_5$, alkali-metal acetates, malic acid, and H_2O_2. The calcination temperatures for each material are listed in Table 1.

Characterization. X-ray diffraction (XRD) patterns were recorded using a Rigaku MiniFlex 600 (tube voltage: 40 kV, tube current: 15 mA) equipped with a Cu Kα radiation source. The samples were scanned four times in the range $10° \leq 2\theta \leq 90°$ at 20° min^{-1} and with a resolution of 0.02°. The Brunauer–Emmett–Teller (BET) specific surface area was estimated from N_2 isotherms obtained using a TriStar II 3020 (Micromeritics, U.S.A.) at 77 K. The analyzed samples were evacuated at 423 K for 3 h prior to the measurement. Thermogravimetry–differential thermal analysis (TG–DTA) profiles were recorded with a DTG-60 (Shimadzu, Japan). Samples (5 mg) were measured by TG–DTA under flowing air or N_2 (200 mL min^{-1}). Inductively coupled plasma–

atomic emission spectroscopy (ICP–AES) analyses were performed with a ICPS-8100 spectrometer (Shimadzu, Japan). Solid materials were pretreated with aqua regia and aqueous HF under microwave irradiation using a Multiwave 3000 (Anton Paar Japan, Japan) before the ICP–AES measurements. The morphology of the samples was examined by scanning electron microscopy (SEM; S-5500, Hitachi, Japan). Transmission electron microscopy (TEM) images were acquired using a JEM2100F (JEOL, Japan) operated at an accelerating voltage of 200 kV. Samples were suspended in ethanol and dropped onto Cu grids, followed by evaporation of the ethanol. X-ray photoelectron spectroscopy (XPS) analysis of the catalysts was performed using a ESCA-3200 spectrometer (Shimadzu, Japan). The spectra were acquired using Al Kα radiation. All spectra were calibrated using C 1*s* (284.5 eV) as a reference. Spectra were deconvoluted with some functions and a Shirley function. X-ray adsorption spectroscopy (XAS) analysis of the catalysts was performed at the BL01B1 beamline at SPring-8 (Japan Synchrotron Radiation Research Institute, Hyogo, Japan). The ring energy was 8 GeV, and the stored current was 99.5 mA. Ti *K*-edge (4.96 keV) X-ray absorption spectra were recorded using a Si(111) double-crystal monochromator. All spectra were recorded using the transmission method in quick-scan mode with ion chambers as detectors. Data reduction was performed using xTunes (Science & Technology Instruments).[S3] FT-IR spectra were recorded at 298 K using an FT/IR-6600 (JASCO, Japan) equipped with a mercury–cadmium–telluride (MCT) detector with a resolution of 4 cm^{-1}. A total of 64 scans were averaged for each spectrum. Each catalyst (30 mg) was pressed into a self-supporting wafer (diameter 20 mm). The catalysts were evacuated at 573 K for 1.5 h before probe molecules were introduced. Pyridine (0.5 kPa) was introduced at 298 K for 15 min, followed by evacuation at 423 K for 15 min to remove weakly adsorbed and hydrogen-bonded pyridine species on the catalyst surface. The catalysts were exposed to $CHCl_3$ (0.5 kPa) at 298 K for 15 min.

Acetophenone (0.02 kPa) was introduced at 298 K for 15 min followed by evacuation at 298 K for 1.5 h to remove weakly adsorbed species. All probe molecules were purified before FT-IR spectra were collected. NH_3 and CO_2 temperature-programmed desorption (NH_3-TPD and CO_2-TPD, respectively) was carried out using a BELCAT A (MicrotracBEL, Japan). The sample (100 mg) was pretreated under He at 773 K for 1 h. Adsorption of probe molecules was carried out for 30 min with 5% NH_3/He or 10% CO_2/He at 373 K, followed by purging with He for 15 min. The temperature was linearly increased from 373 to 873 K at 10 K min^{-1}. The outlet flow was analyzed using a thermal conductivity detector (TCD) or Q-Mass (BELMASS, MicrotracBEL, Japan). Gas chromatography (GC) analysis was performed using a GC-2025 (Shimadzu, Japan) equipped with a flame ionization detector and an InertCap 5 capillary column (length: 60 m, internal diameter: 0.25 mm, film thickness: 0.40 μm, GL Sciences, Japan). GC–mass spectrometry (GC–MS) analysis was performed using a GCMS-QP2010 SE (Shimadzu, Japan) operated at an ionization voltage of 70 eV and equipped with an InertCap 17MS capillary column (length: 30 m, internal diameter: 0.25 mm, film thickness: 0.25 μm, GL Sciences, Japan). Nuclear magnetic resonance (NMR) spectra were recorded on a Bruker Biospin Avance III spectrometer (FT, 400 MHz (^{1}H), 100 MHz (^{13}C)). Chemical shifts (δ) of the ^{1}H and ^{13}C NMR spectra were referenced to $SiMe_4$. The crystal structures in the present paper were drawn using the VESTA ver. 3.5.6 software.[S4,S5]

Catalytic reaction. A typical procedure for cyanosilylation was as follows: Catalyst (50 mg), carbonyl compounds (1 mmol), TMSCN (1.5 mmol), toluene (2 mL), and *n*-decane (0.5 mmol) as an internal standard were added to a reactor under an Ar atmosphere. The reaction mixture was stirred at 275 K in an ice bath and 298 K in an organic synthesizer. After the reaction was completed, the catalysts were collected by filtration, washed with toluene (25 mL), water (25 mL),

and MeOH (25 mL), dried at 373 K for 6 h, and calcined at 823 K for 1 h to remove residues from catalyst surface prior to being recycled. The recovered catalysts even without heat treatment could be reused to give **2a** in 91% yield comparable to that (99%) of the fresh catalyst. The products were collected after evaporation of the filtrate and distillation using a kugelrohr apparatus.

Data of products

2-phenyl-2-((trimethylsilyl)oxy)propanenitrile (**2a**)[S6]:

^{1}H NMR (400 MHz, $CDCl_3$, ppm): δ 0.17 (s, 9H), 1.85 (s, 3H), 7.30–7.44 (m, 3H), 7.53–7.56 (m, 2H). ^{13}C NMR (100 MHz, $CDCl_3$, ppm): δ 1.00, 33.50, 121.58, 124.56, 128.58, 128.59, 141.95.

2-(*o*-tolyl)-2-((trimethylsilyl)oxy)propanenitrile (**2b**)[S7]:

^{1}H NMR (400 MHz, $CDCl_3$, ppm): δ 0.19 (s, 9H), 1.96 (s, 3H), 2.57 (s, 3H), 7.18–7.28 (m, 3H), 7.59 (d, J= 7.8 Hz, 1H). ^{13}C NMR (100 MHz, $CDCl_3$, ppm): δ 1.15, 20.74, 30.58, 71.74, 121.69, 125.35, 126.02, 128.71, 132.70, 135.09, 135.58.

2-(*m*-tolyl)-2-((trimethylsilyl)oxy)propanenitrile (**2c**)[S8]:

^{1}H NMR (400 MHz, $CDCl_3$, ppm): δ 0.17 (s, 9H), 1.84 (s, 3H), 2.39 (s, 3H), 7.15–7.16 (m, 1H), 7.26–7.29 (m, 1H), 7.33–7.34 (m, 2H). ^{13}C NMR (100 MHz, $CDCl_3$, ppm): δ 1.09, 21.50, 33.57, 71.65, 121.69, 125.30, 128.08, 128.35, 129.37, 138.40, 141.95.

2-(*p*-tolyl)-2-((trimethylsilyl)oxy)propanenitrile (**2d**)[S9]:

^1H NMR (400 MHz, $CDCl_3$, ppm): δ 0.16 (s, 9H), 1.84 (s, 3H), 2.36 (s, 3H), 7.19 (d, *J*= 8.0 Hz, 2H), 7.42 (d, *J*= 8.2 Hz, 2H). ^{13}C NMR (100 MHz, $CDCl_3$, ppm): δ 1.08, 20.07, 33.53, 71.53, 121.78, 124.60, 129.26, 138.54, 139.10.

2-(4-methoxyphenyl)-2-((trimethylsilyl)oxy)propanenitrile (**2e**)[S10]:

^1H NMR (400 MHz, $CDCl_3$, ppm): δ 0.15 (s, 9H), 1.84 (s, 3H), 3.82 (s, 3H), 6.91 (d, *J*= 8.8 Hz, 2H), 7.46 (d, *J*= 8.8 Hz, 2H). ^{13}C NMR (100 MHz, $CDCl_3$, ppm): δ 1.08, 33.42, 55.35, 71.28, 113.89, 121.81, 126.06, 134.07, 159.80.

2-(4-chlorophenyl)-2-((trimethylsilyl)oxy)propanenitrile (**2f**)[S9]:

^1H NMR (400 MHz, $CDCl_3$, ppm): δ 0.19 (s, 9H), 1.83 (s, 3H), 7.36–7.38 (m, 2H), 7.47–7.49 (m, 2H). ^{13}C NMR (100 MHz, $CDCl_3$, ppm): δ 1.06, 33.53, 71.08, 121.26, 126.09, 128.85, 134.62, 140.73.

2-(4-bromophenyl)-2-((trimethylsilyl)oxy)propanenitrile (**2g**)[S9]:

^1H NMR (400 MHz, $CDCl_3$, ppm): δ 0.19 (s, 9H), 1.83 (s, 3H), 2.28 (s, 3H), 7.42 (d, *J*= 8.6, 2H), 7.53 (d, *J*= 8.6, 2H). ^{13}C NMR (100 MHz, $CDCl_3$, ppm): δ 1.07, 33.51, 71.13, 121.19, 122.75, 126.39, 131.82, 141.28.

2-(4-(trifluoromethyl)phenyl)-2-((trimethylsilyl)oxy)propanenitrile (**2h**):

^1H NMR (400 MHz, $CDCl_3$, ppm): δ 0.21 (s, 9H), 1.86 (s, 3H), 7.65–7.70 (m, 4H). ^{13}C NMR (100 MHz, $CDCl_3$, ppm): δ 1.05, 33.56, 71.13, 121.03, 125.08, 125.74, 125.77, 146.06.

2-(4-nitrophenyl)-2-((trimethylsilyl)oxy)propanenitrile (**2i**)[S6]:

^{1}H NMR (400 MHz, $CDCl_3$, ppm): δ 0.24 (s, 9H), 1.88 (s, 3H), 7.74 (d, *J*= 8.9 Hz, 2H), 8.27 (d, *J*= 8.90, 2H). ^{13}C NMR (100 MHz, $CDCl_3$, ppm): δ 1.06, 33.48, 70.93, 120.64, 124.01, 125.73, 148.08, 148.98.

2-cyclopropyl-2-phenyl-2-((trimethylsilyl)oxy)acetonitrile (**2j**):

^{1}H NMR (400 MHz, $CDCl_3$, ppm): δ 1.34 (s, 9H), 0.55–0.63 (m, 2H), 0.64–0.87 (m, 2H), 1.34–1.41 (m, 1H), 7.33–7.41 (m, 3H), 7.54–7.57 (m, 2H). ^{13}C NMR (100 MHz, $CDCl_3$, ppm): δ 0.99, 2.59, 2.88, 24.02, 75.47, 120.21, 125.25, 128.48, 128.69, 141.37.

2,2-diphenyl-2-((trimethylsilyl)oxy)acetonitrile (**2k**)[S6]:

^{1}H NMR (400 MHz, $CDCl_3$, ppm): δ 0.13 (s, 9H), 7.29–7.38 (m, 6H), 7.49–7.51 (m, 4H). ^{13}C NMR (100 MHz, $CDCl_3$, ppm): δ 0.91, 76.38, 120.73, 125.93, 128.57, 128.67, 141.98.

9-((trimethylsilyl)oxy)-9H-fluorene-9-carbonitrile (**2l**)[S6]:

^{1}H NMR (400 MHz, $CDCl_3$, ppm): δ 0.08 (s, 9H), 7.64 (t, *J*= 8.0 Hz, 2H), 7.73 (t, *J*= 8.0 Hz, 2H), 7.90 (d, *J*= 7.6 Hz, 2H), 7.99 (d, *J*= 7.5 Hz, 2H). ^{13}C NMR (100 MHz, $CDCl_3$, ppm): δ 1.23, 120.54, 125.48, 128.77, 130.93, 139.53, 143.07.

1-((trimethylsilyl)oxy)cyclohexane-1-carbonitrile (**2m**)[S11]:

^{1}H NMR (400 MHz, $CDCl_3$, ppm): δ 0.24 (s, 9H), 1.18–1.30 (m, 1H), 1.50–1.63 (m, 5H), 1.71–1.78 (m, 2H), 2.03–2.07 (m, 2H). ^{13}C NMR (100 MHz, $CDCl_3$, ppm): δ 1.44, 22.66, 24.55, 39.38, 70.67, 122.00.

(1r,3r,5r,7r)-2-((trimethylsilyl)oxy)adamantane-2-carbonitrile (**2n**)[S12,S13]:

^{1}H NMR (400 MHz, $CDCl_3$, ppm): δ 0.26 (s, 9H), 1.54–2.12 (m, 14H). ^{13}C NMR (100 MHz, $CDCl_3$, ppm): δ 1.28, 26.21, 26.39, 30.98, 34.76, 37.24, 38.13, 74.75, 122.16.

2-methyl-2-((trimethylsilyl)oxy)octanenitrile (**2o**)[S14]:

^{1}H NMR (400 MHz, $CDCl_3$, ppm): δ 0.23 (s, 9H), 0.89 (t, *J*= 6.7 Hz, 3H), 1.30–1.36 (m, 6H), 1.38–1.53 (m, 2H), 1.56 (s, 3H), 1.64–1.77 (m, 2H). ^{13}C NMR (100 MHz, $CDCl_3$, ppm): δ 1.31, 14.02, 22.53, 24.24, 28.91, 28.99, 31.62, 43.41, 69.71, 122.24.

2,4-dimethyl-2-((trimethylsilyl)oxy)pentanenitrile (**2p**)[S14]:

^{1}H NMR (400 MHz, $CDCl_3$, ppm): δ 0.24 (s, 9H), 1.00 (dd, *J*= 6.7; 4.0, 6H), 1.58–1.69 (m, 5H), 1.87–2.00 (m, 1H). ^{13}C NMR (100 MHz, $CDCl_3$, ppm): δ 1.33; 23.69; 23.80; 24.93; 29.81; 51.56; 69.18; 122.58.

2-phenyl-2-((trimethylsilyl)oxy)acetonitrile (**2q**)[S15]:

^{1}H NMR (400 MHz, $CDCl_3$, ppm): δ 0.23 (s, 9H), 5.50 (s, 1H), 7.38–7.48 (m, 5H). ^{13}C NMR (100 MHz, $CDCl_3$, ppm): δ –0.24, 63.70, 119.18, 126.38, 128.96, 129.35, 136.31.

Table S1. Synthesis method and properties of reported $SrTiO_3$

entry	method	calcination conditions	grain size / nm	particle size / nm	S_{BET} / m^2 g^{-1}	remarks	ref.
1	Sol–gel (DL-Malic acid)	823 K, 5 h, N_2→air	23	10–30	46	-	This work
2	Sol–gel (DL-Malic acid)	823 K, 5 h, air	31	30–40	30	-	This work
3	Solid-state	1523 K, 3.5 h, air	-	1000	2	-	S16
4	Molten salt	1093 K, 3.5 h, air	-	120	10	0.6% $SrCO_3$ was contained as impurity.	S16
5	Co-precipitation	973 K	34	-	20	2% TiO_2 was contained as impurity.	S17
7	Sol–gel	973 K	-	35	20	-	S18
8	Sol–gel	923 K	-	5–13	46	$TiOCl_2$ was used as precursor.	S19
9	Polymerized complex (Citric acid + EG)	1073 K	-	31	20	-	S20

10	Polymerized complex (Citric acid + EG)	Pyrolysis 1023 K, 2h, N_2 → 673 K, 4 h, air	-	11	83	0.4% $SrCO_3$ was contained as impurity.	S21
11	Hydrothermal	513 K, 36 h	-	60	20	0.4% $SrCO_3$ was contained as impurity.	S16
12	Hydrothermal (PVA + KOH)	473 K, 12 h	-	21	50	$SrCO_3$ was washed with HNO_3.	S22
13	Solvothermal (MeOH) (KOH + oleic acid)	473 K, 3 h (microwave irradiation)	17		41	-	S23
14	Ball milling	1273 K	-	51	10	-	S24
15	Templating (F127)	1123 K	-	-	86	Not only diffraction patters of cubic perovskite were observed.	S25

Table S2. Cyanosilylation of acetophenone (**1a**) with TMSCN over solid catalysts

entry	catalyst	pretreatment	W_{Cat} / mg	**1a** /mmol	TMSCN / mmol	solvent (V / mL)	temp / K	time / min	yield (%)	rate / mmol $g^{-1}min^{-1}$	ref.
1		573 K, 1 h, *in vacuo*	25	1.0	1.5	Toluene (2)	298	5	90	1.4×10^{1}	
2		-	25	1.0	1.5	Toluene (2)	298	15	> 99	8.4	
3	$SrTiO_3$_N_2-air	-	50	1.0	1.5	neat	298	1	> 99	2.1×10^{1}	This work
4		573 K, 1 h, *in vacuo*	50	1.0	1.5	Toluene (2)	ice bath	5	97	6.3	
5		-	50	1.0	1.5	Toluene (2)	ice bath	30	> 99	2.2	
6	Sn-Mont	393 K, 1 h, *in vacuo*	10	1.0	1.2	CH_2Cl_2 (2)	r.t.	1	98	9.8×10^{1}	S6
7	Al-MCM-41 (Si/Al= 20)	393 K, 1 h, *in vacuo*	5	1.0	1.2	CH_2Cl_2 (2)	r.t.	5	90	3.6×10^{1}	S26
8	nano CeO_2	673 K	20 wt%	0.25	0.3	*n*-Hexane (0.3)	298	60	55	6.9×10^{-1}	S27
9	Sn–W oxide (Sn/W=2)	573 K, 1 h, *in vacuo*	50	1.0	4.0	$C_2H_4Cl_2$ (0.5)	r.t.	30	95	6.7×10^{-1}	S28
10	Fe-Mont	393 K, 3 h, *in vacuo*	200	1.0	1.2	CH_2Cl_2 (4)	273	12	96	4.2×10^{-1}	S29
11	MgO	773 K, 2 h, *in vacuo*	200	1.0	1.2	CH_2Cl_2 (4)	273	108	96	4.6×10^{-2}	S29
12	CaO	773 K, 2 h, *in vacuo*	200	1.0	1.2	CH_2Cl_2 (4)	273	113	93	4.4×10^{-2}	S29

13	Hydroxyapatite	453 K, 2 h, *in vacuo*	500	1.0	1.1	CH_2Cl_2 (4)	273	78	96	2.6×10^{-2}	S30
14	CaF_2	453 K, 2 h, *in vacuo*	500	1.0	1.1	CH_2Cl_2 (4)	273	300	98	6.7×10^{-3}	S30
15	$MgAlCO_3$	-	200	4.0	6.0	Heptane (-)	r.t.	5	97	3.9	S31
16	MNPs-guanidine [a]	-	5	1.0	1.2	CH_2Cl_2 (2)	r.t.	120	85	1.4	S32
17	1-OH/SiO_2 [b]	-	75	0.5	2.0	CH_2Cl_2 (0.5)	305	60	94	1.1×10^{-1}	S33
18	SILLP-7 [c]	-	75	3.0	3.6	neat	r.t.	24 h	92	2.8×10^{-2}	S34
19	diamino-functionalised MCM-41	-	200	1.0	1.5	Toluene (10)	r.t	12 h	28	6.9×10^{-3}	S35
20	$Zr(K_2PO_4)_2$	-	200	2.0	3.0	CH_2Cl_2 (2)	reflux	24 h	98	6.9×10^{-3}	S36
21	Mg_3Al–EuW_{10} [d]	-	0.25 mol%	1.0	1.5	neat	298	6 h	95	-	S37
22	Al-ITQ-HB [e]	-	1 mol%	5.08	6.10	neat	r.t.	180	99	-	S38
23	Triphenylphosphine /polystyrene	-	2 mol%	0.5	0.6	neat	333	180	97	-	S14
24	In(IDA)(Cl) [f]		1 mol%	1.0	1.1	-	298	240	96	-	S39

[a] Guanidine immobilized on magnetic nanoparticle (Fe_3O_4). [b] N-octyldihy-droimidazolium hydroxide fragment onto SiO_2. [c] Ionic liquid-like materials supported on PS-DVB polymer. [d] Europium-containing polyoxotungstate intercalated into layered double hydroxides (LDHs). [e] Metalorganic hybrid supramolecular between chained AlO_6 and 4-heptylbenzoic acid. [f] Inorganic–organic hybrid compounds constructed with $InCl_3$ and iminodiacetic acid.

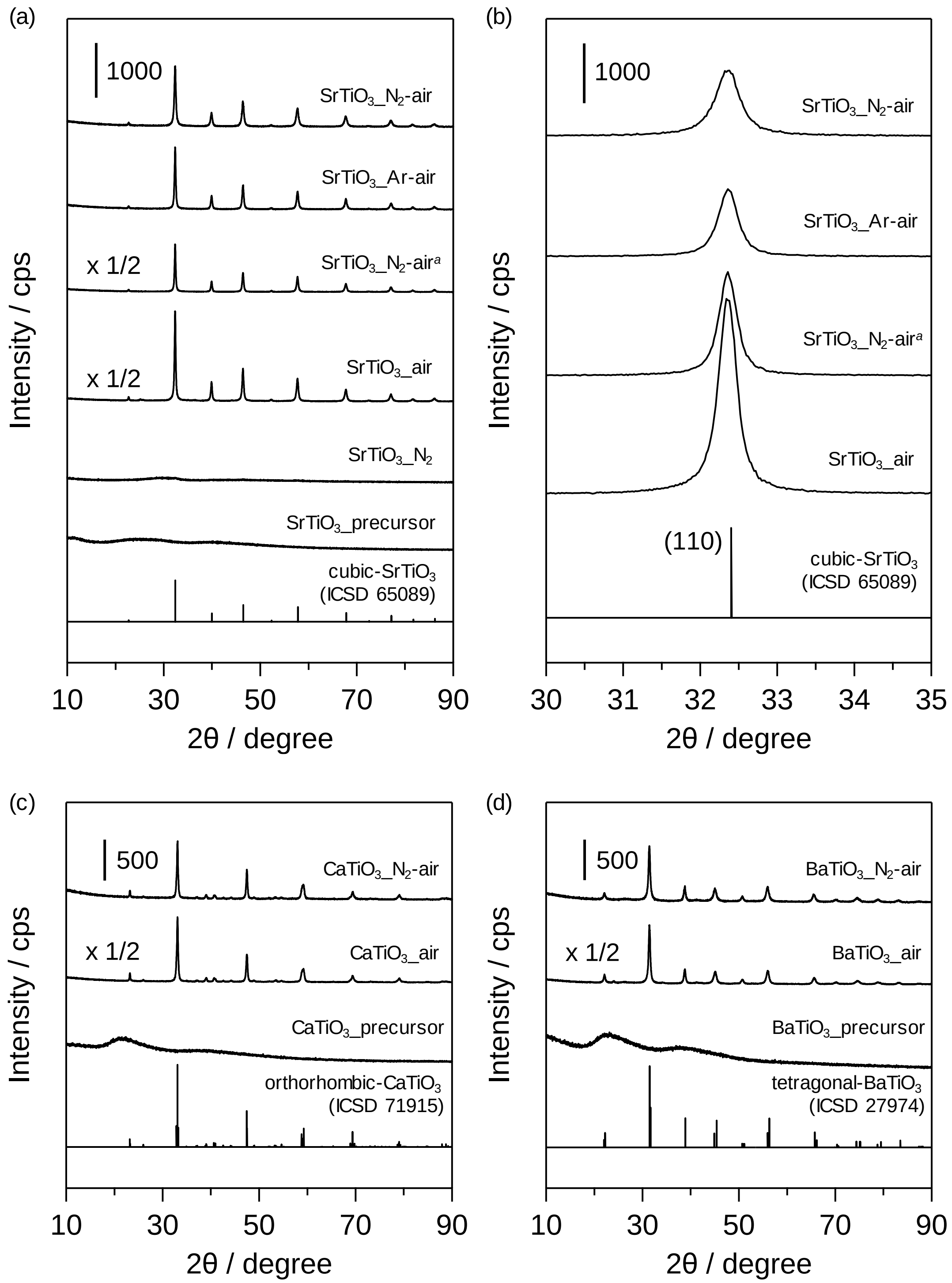

Figure S1. (a, c, d) XRD patterns for Ti-based perovskite oxides. (b) XRD patterns for $SrTiO_3$ focused on (110) face. [a]: $SrTiO_3$_N_2-air synthesized using citric acid instead of malic acid.

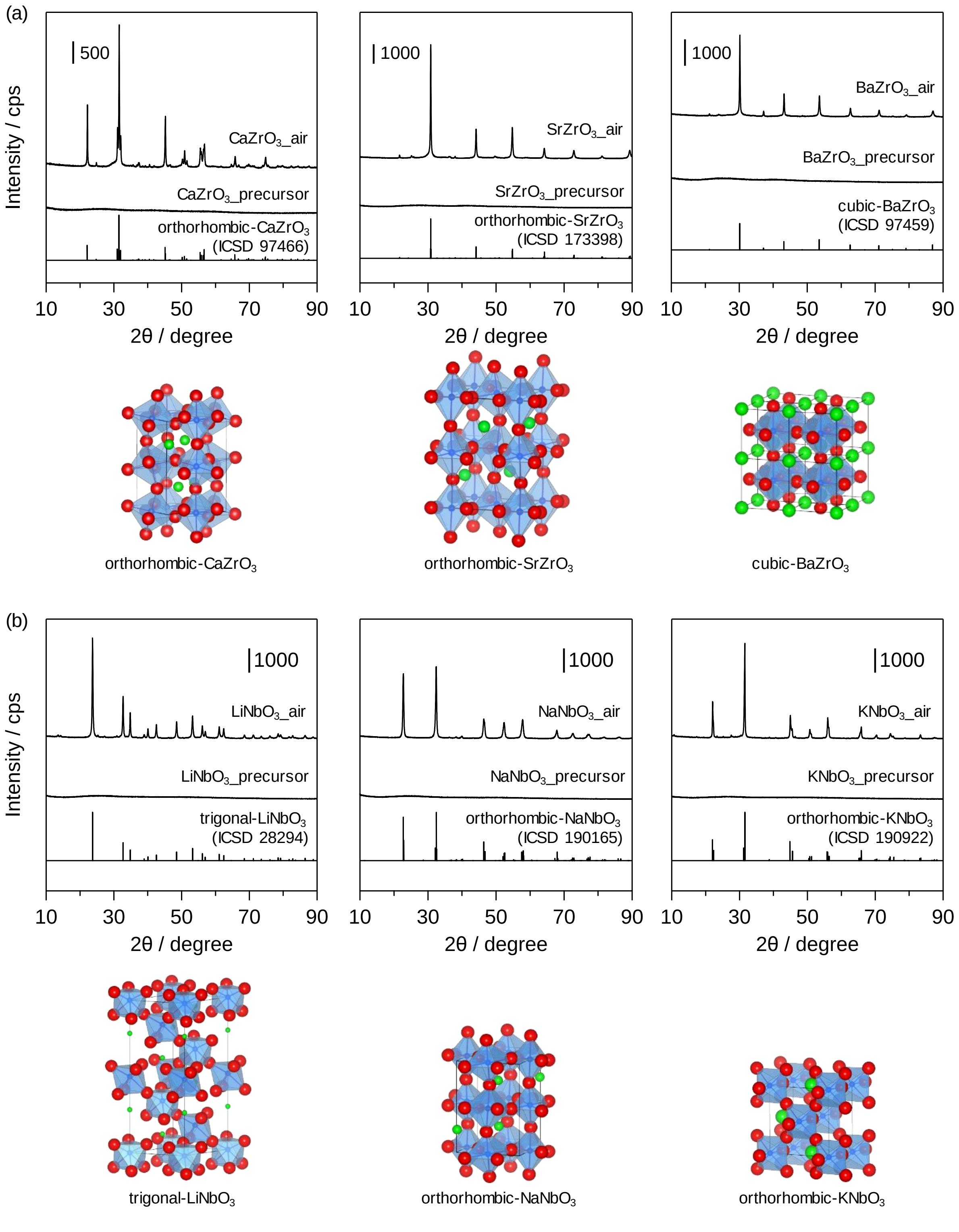

Figure S2. XRD patterns for (a) Zr- and (b) Nb-based perovskite oxides and their structures. Green, blue, and red spheres represent *A*-site cations, *B*-site cations, and O^{2-} anions, respectively.

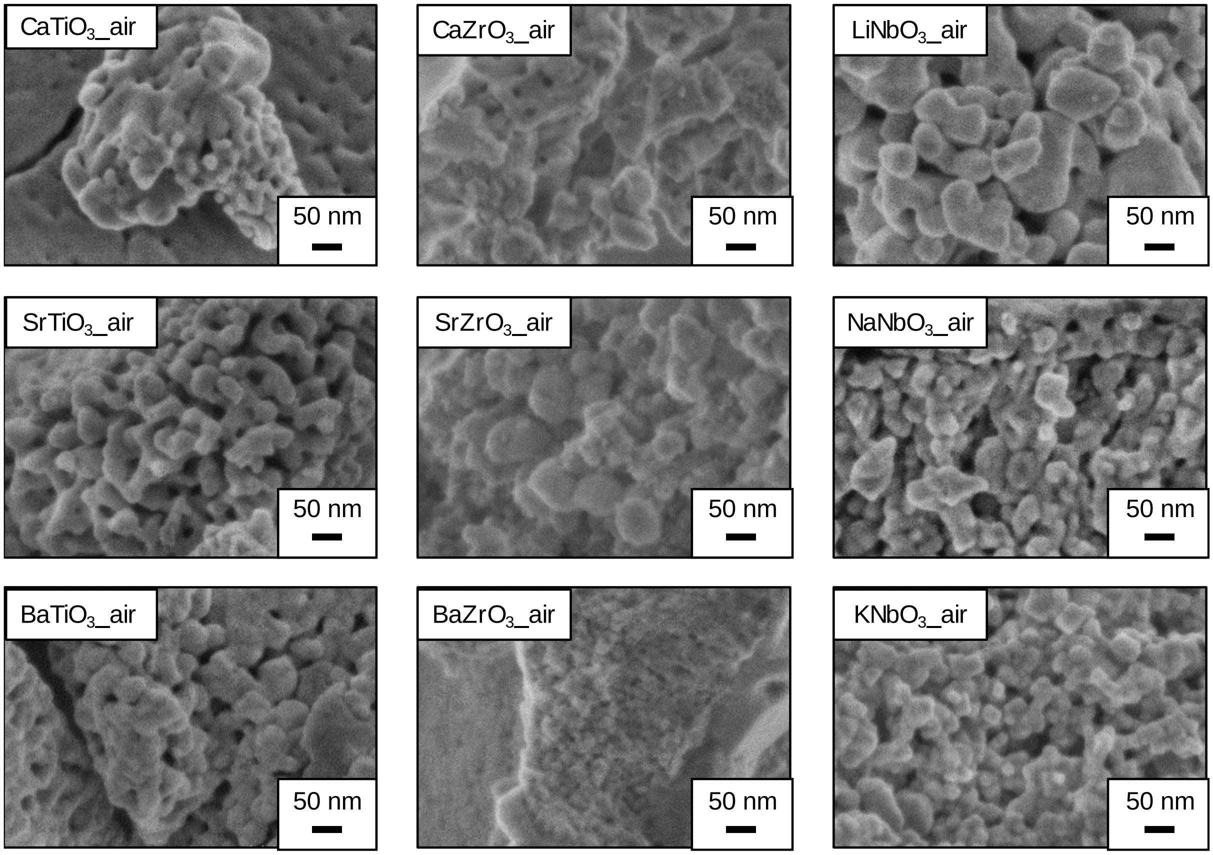

Figure S3. SEM images for d^0-transition-metal-cation-based perovskite oxides ABO$_3$_air.

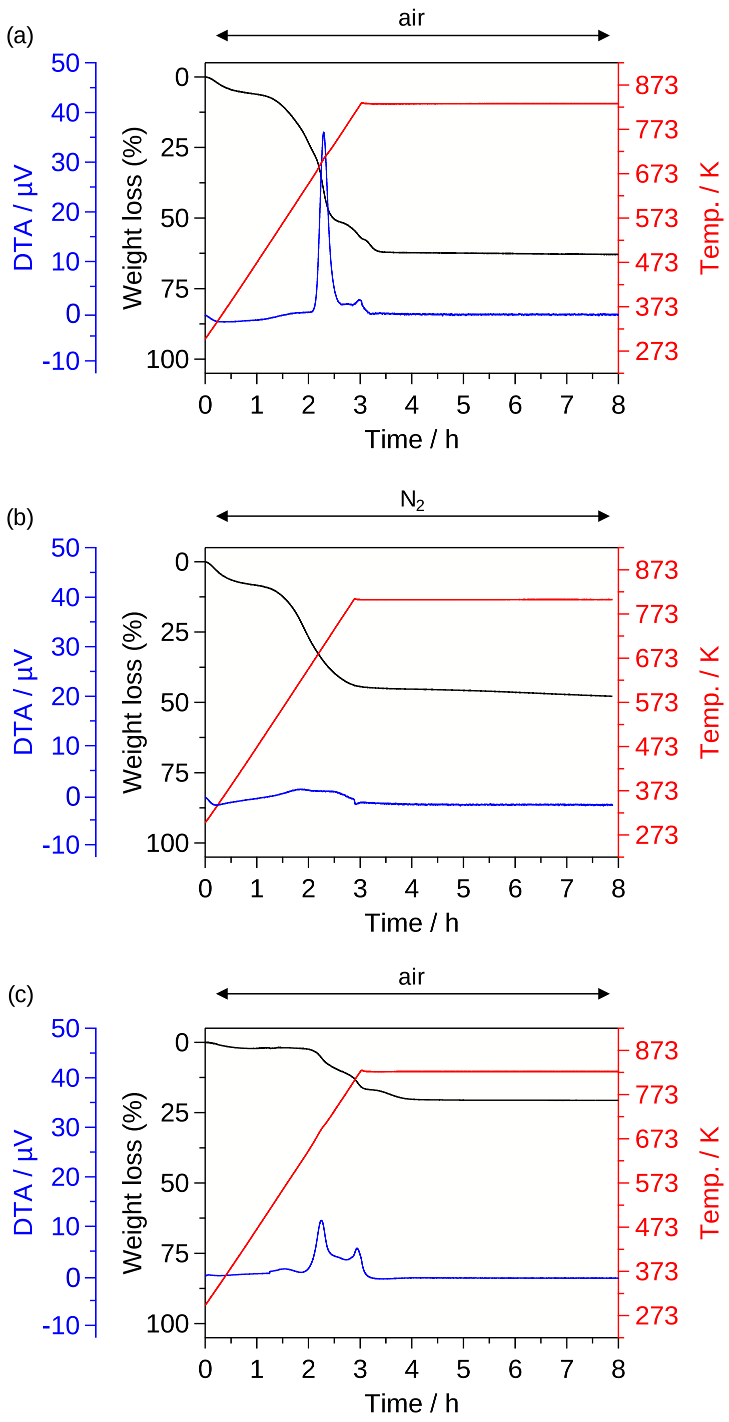

Figure S4. TG–DTA profiles for $SrTiO_3$ precursor under (a) air and (b) N_2 atmospheres. (c) Profiles under air for $SrTiO_3$ precursor treated at 823 K for 5 h under N_2 before TG–DTA measurement.

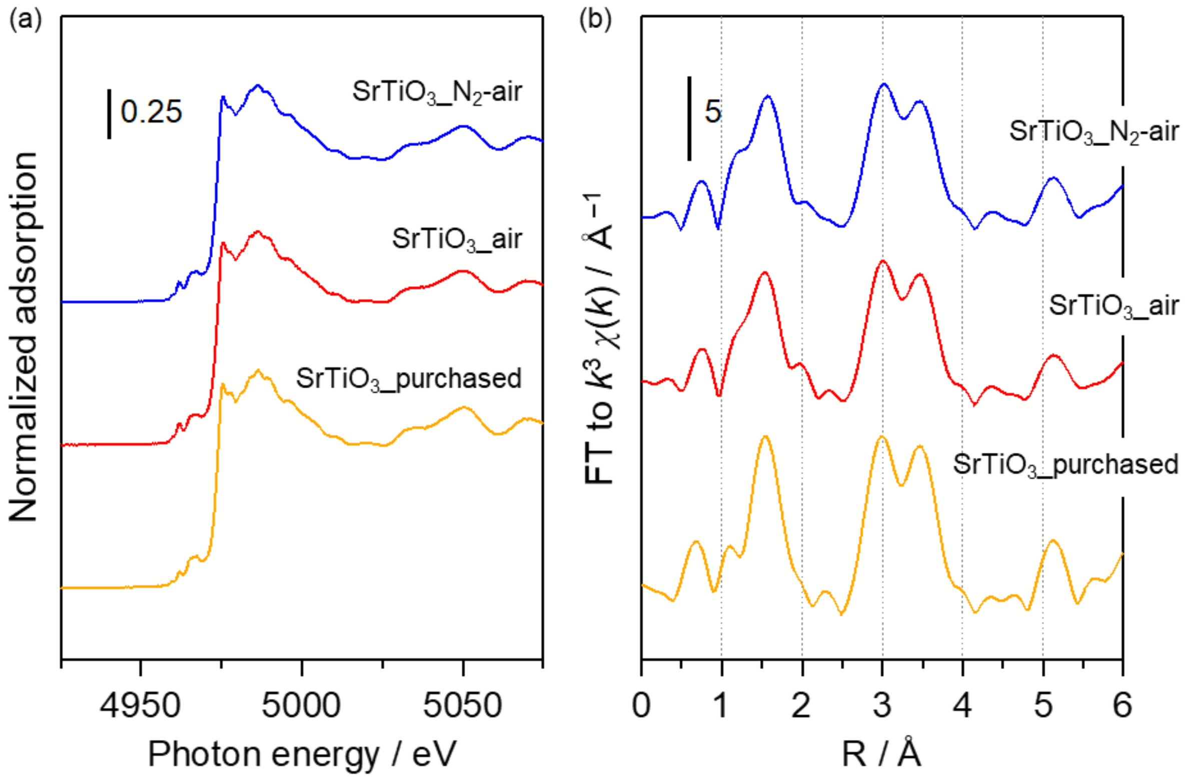

Figure S5. (a) Ti *K*-edge XAFS spectra and (b) Fourier-transformed Ti-edge EXAFS oscillations for $SrTiO_3$.

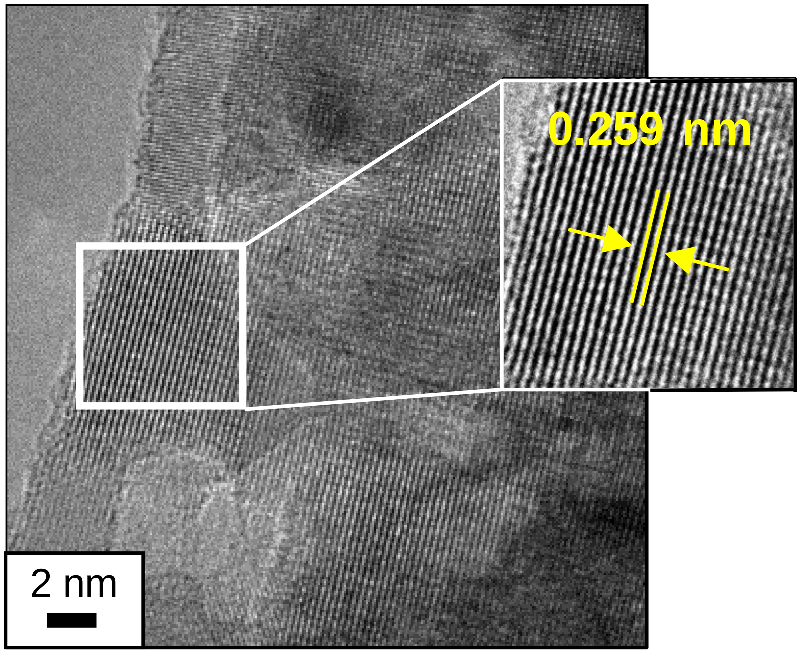

Figure S6. TEM image of $SrTiO_3$_air.

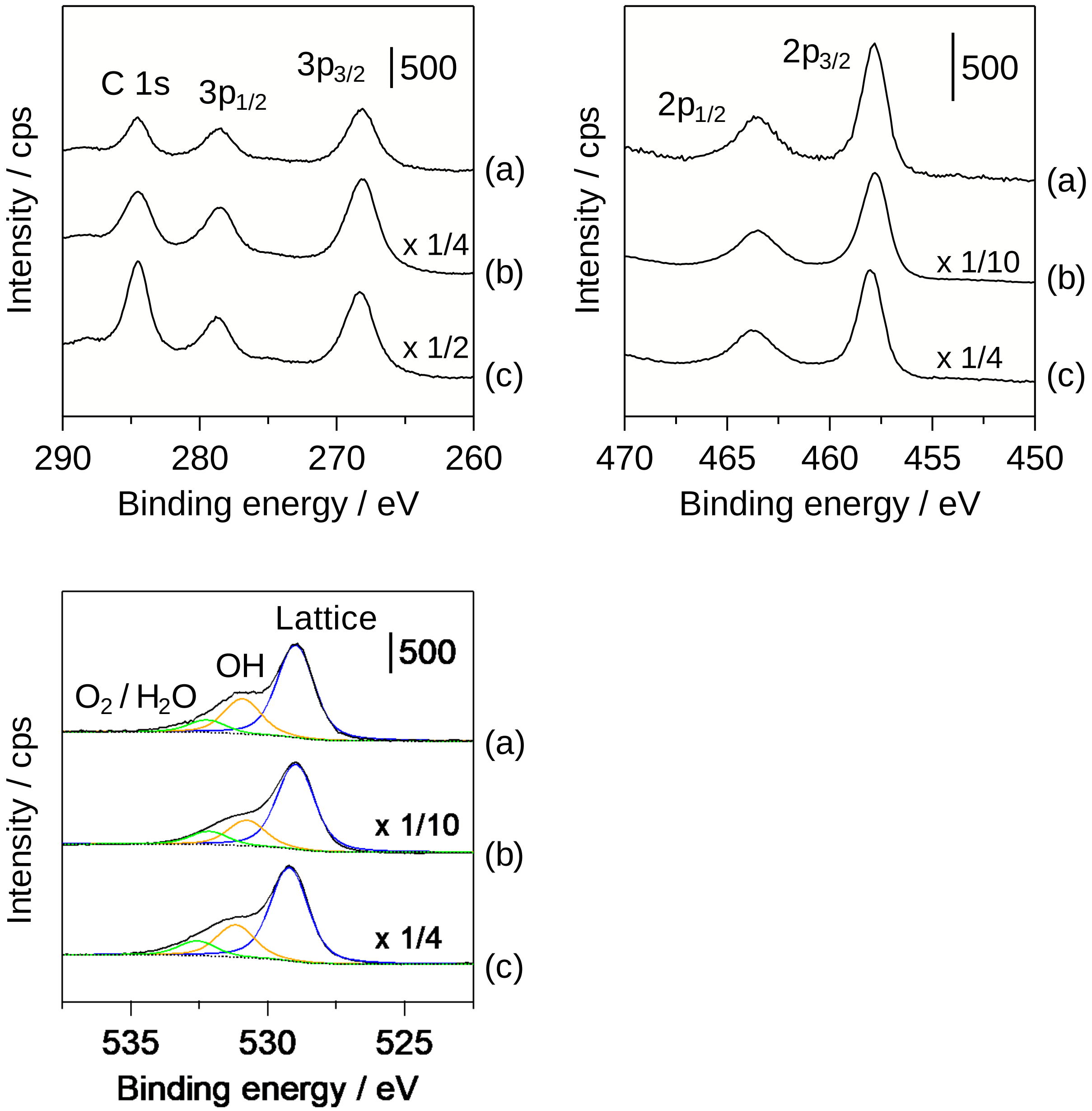

Figure S7. XP spectra of (a) $SrTiO_3$_N_2-air, (b) $SrTiO_3$_air, and (c) $SrTiO_3$_purchased.

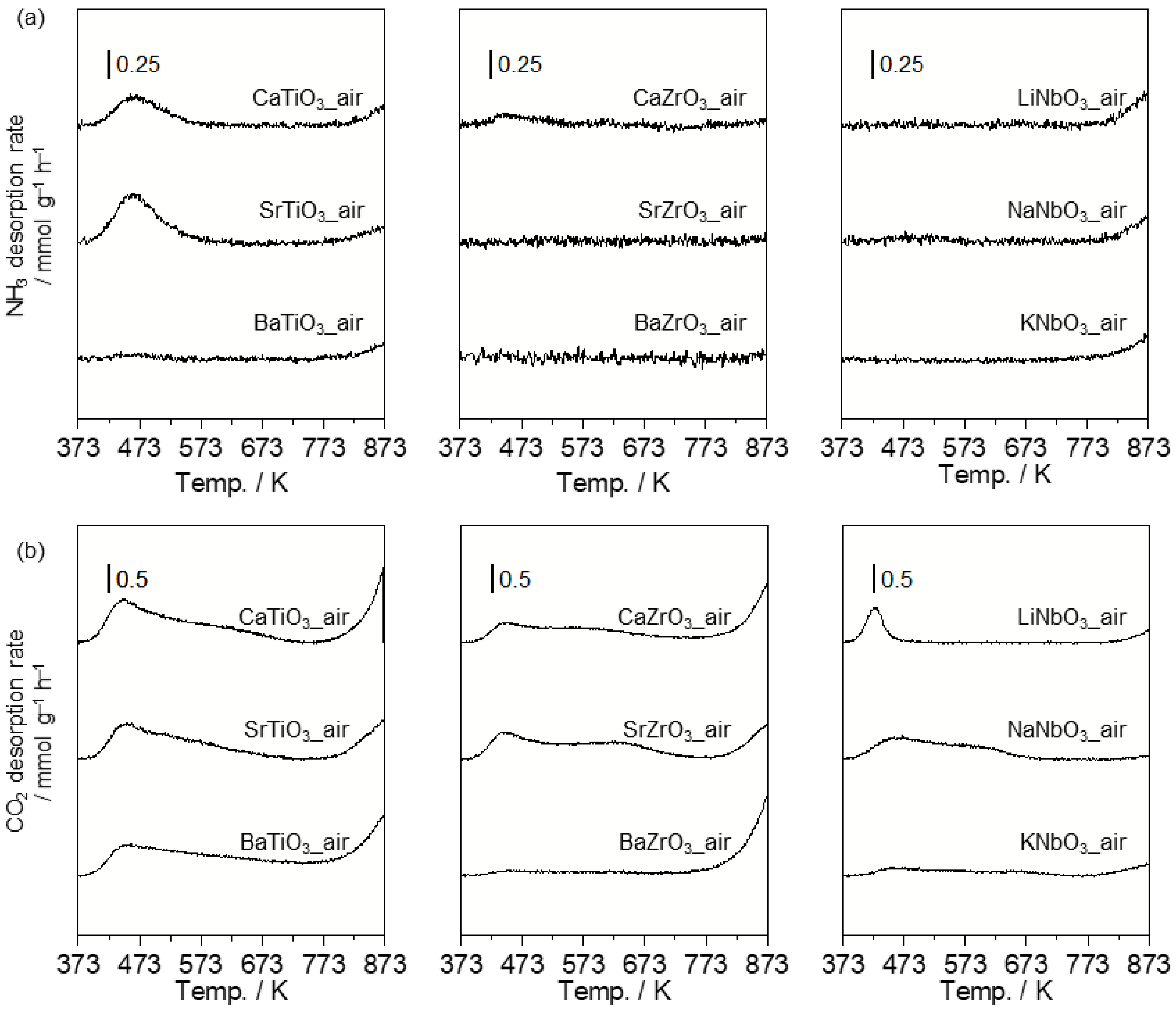

Figure S8. (a) NH_3- and (b) CO_2-TPD profiles for perovskite oxides with d^0-transition-metal *B*-site cations Ti^{4+}, Zr^{4+}, and Nb^{5+}.

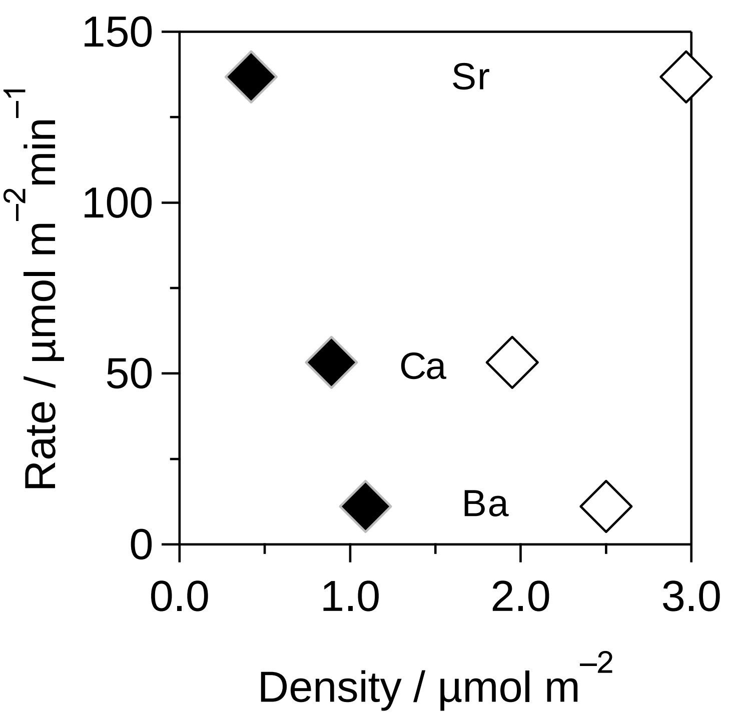

Figure S9. Relationship between density of acid (◆) and base (◇) sites and cyanosilylation rate over Ti-based perovskite oxides. Reaction conditions: Catalyst (50 mg), **1a** (1.0 mmol), TMSCN (1.5 mmol), toluene (2 mL), ice bath (275 K), Ar atmosphere. Catalysts were pretreated at 573 K for 1 h *in vacuo*.

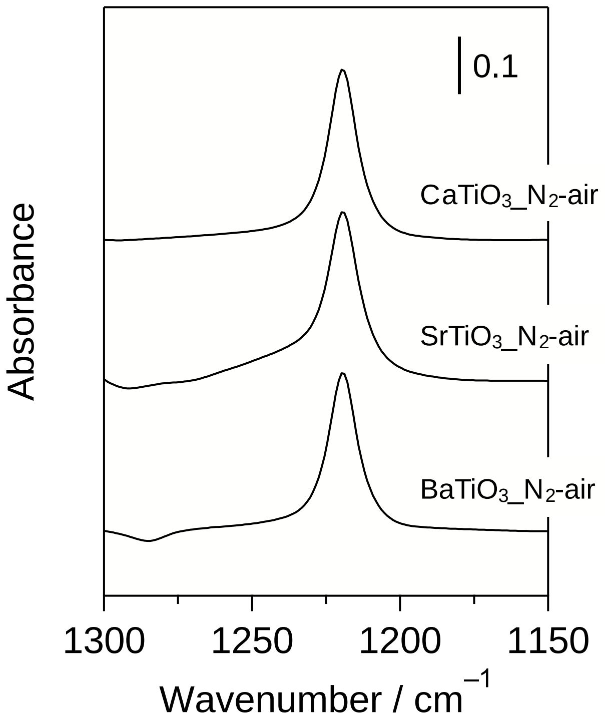

Figure S10. Difference FT-IR spectra of $CHCl_3$ in region adsorbed onto $A$$TiO_3$_$N_2$-air.

,

References

(S1) Williams, D. B. G.; Lawton, M. Drying of Organic Solvents: Quantitative Evaluation of the Efficiency of Several Desiccants. *J. Org. Chem.* 2010, *75*, 8351–8354, 10.1021/jo101589h.

(S2) Evans, D. A.; Carroll, G. L.; Truesdale, L. K. Synthetic Applications of Trimethylsilyl Cyanide. Efficient Synthesis of β-Aminomethyl Alcohols. *J. Org. Chem.* 1974, *39*, 914–917, 10.1021/jo00921a012.

(S3) Asakura, H.; Yamazoe, S.; Misumi, T.; Fujita, A.; Tsukuda, T.; Tanaka, T. XTunes: A New XAS Processing Tool for Detailed and on-the-Fly Analysis. *Radiat. Phys. Chem.* 2020, *175*, 108270, 10.1016/j.radphyschem.2019.04.020.

(S4) Momma, K.; Izumi, F. VESTA: a three-dimensional visualization system for electronic and structural analysis. *J. Appl. Crystallogr.* 2008, *41*, 653–658, 10.1107/S0021889808012016.

(S5) Momma, K.; Izumi, F. VESTA 3 for Three-Dimensional Visualization of Crystal, Volumetric and Morphology Data. *J. Appl. Phys.* 2011, *44*, 1272–1276, 10.1107/S0021889811038970.

(S6) Wang, J.; Masui, Y.; Watanabe, K.; Onaka, M. Highly Efficient Cyanosilylation of Sterically Bulky Ketones Catalyzed by Tin Ion-Exchanged Montmorillonite. *Adv. Synth. Catal.* 2009, *351*, 553–557, 10.1002/adsc.200800673.

(S7) Fuerst, D. E.; Jacobsen, E. N. Thiourea-Catalyzed Enantioselective Cyanosilylation of Ketones. *J. Am. Chem. Soc.* 2005, *127*, 8964–8965, 10.1021/ja052511x.

(S8) Zhou, H.; Zhou, Y.; Bae, H. Y.; Leutzsch, M.; Li, Y.; De, C. K.; Cheng, G.-J.; List, B. Organocatalytic Stereoselective Cyanosilylation of Small Ketones. *Nature* 2022, *605*, 84–89, 10.1038/s41586-022-04531-5.

(S9) Mei, L.; Long, S. W.; Liang, H. K.; Xuan, W. S. Studies on Cyanosilylation Reaction Catalyzed by Ln–N Complexes. *Appl. Organomet. Chem.* 2008, *22*, 181–186, 10.1002/aoc.1369.

(S10) Deng, H.; Isler, M. P.; Snapper, M. L.; Hoveyda, A. H. Aluminum-Catalyzed Asymmetric Addition of TMSCN to Aromatic and Aliphatic Ketones Promoted by an Easily Accessible and Recyclable Peptide Ligand. *Angew. Chem., Int. Ed.* 2002, *41*, 1009–1012, 10.1002/1521-3773(20020315)41:6<1009::AID-ANIE1009>3.0.CO;2-F.

(S11) Lacour, M. A.; Rahier, N. J.; Taillefer, M. Mild and Efficient Trimethylsilylcyanation of Ketones Catalysed by PNP Chloride. *Chem. – Eur. J.* 2011, *17*, 12276–12279, 10.1002/chem.201101195.

(S12) Kolocouris, A.; Koch, A.; Kleinpeter, E.; Stylianakis, I. 2-Substituted and 2,2-Disubstituted Adamantane Derivatives as Models for Studying Substituent Chemical Shifts and C–$H_{ax}\cdots Y_{ax}$ Cyclohexane Contacts—Results from Experimental and Theoretical NMR Spectroscopic Chemical Shifts and DFT Structures. *Tetrahedron* 2015, *71*, 2463–2481, 10.1016/j.tet.2015.01.044.

(S13) Picard, J. P.; Elyusufi, A. A.; Calas, R.; Dunogues, J.; Duffaut, N. Enamines of Acylsilanes: An Easy Access from *α*-Siloxynitriles. *Organometallics* 1984, *3*, 1660–1665, 10.1021/om00089a011.

(S14) Strappaveccia, G.; Lanari, D.; Gelman, D.; Pizzo, F.; Rosati, O.; Curini, M.; Vaccaro, L. Efficient Synthesis of Cyanohydrin Trimethylsilyl Ethers via 1,2-Chemoselective Cyanosilylation of Carbonyls. *Green Chem* 2013, *15*, 199–204, 10.1039/C2GC36442E.

(S15) Kurono, N.; Yamaguchi, M.; Suzuki, K.; Ohkuma, T. Lithium Chloride: An Active and Simple Catalyst for Cyanosilylation of Aldehydes and Ketones. *J. Org. Chem.* 2005, *70*, 6530–6532, 10.1021/jo050791t.

(S16) Rabuffetti, F. A.; Stair, P. C.; Poeppelmeier, K. R. Synthesis-Dependent Surface Acidity and Structure of $SrTiO_3$ Nanoparticles. *J. Phys. Chem. C* 2010, *114*, 11056–11067, 10.1021/jp101727c.

(S17) Amores, J. M. G.; Escribano, V. S.; Daturi, M.; Busca, G. Preparation, Characterization and Surface Structure of Coprecipitated High-Area Sr_xTiO_{2+x} ($0<x<1$) Powders. *J. Mater. Chem.* 1996, *6*, 879–886, 10.1039/JM9960600879.

(S18) Puangpetch, T.; Sreethawong, T.; Yoshikawa, S.; Chavadej, S. Synthesis and Photocatalytic Activity in Methyl Orange Degradation of Mesoporous-Assembled $SrTiO_3$ Nanocrystals Prepared by Sol–Gel Method with the Aid of Structure-Directing Surfactant. *J. Mol. Catal. Chem.* 2008, *287*, 70–79, 10.1016/j.molcata.2008.02.027.

(S19) Ahuja, S.; Kutty, T. R. N. Nanoparticles of $SrTiO_3$ Prepared by Gel to Crystallite Conversion and Their Photocatalytic Activity in the Mineralization of Phenol. *J. Photochem. Photobiol. Chem.* 1996, *97*, 99–107, 10.1016/1010-6030(96)04324-9.

(S20) García-López, E.; Marcì, G.; Megna, B.; Parisi, F.; Armelao, L.; Trovarelli, A.; Boaro, M.; Palmisano, L. $SrTiO_3$-Based Perovskites: Preparation, Characterization and Photocatalytic

Activity in Gas–Solid Regime under Simulated Solar Irradiation. *J. Catal.* 2015, *321*, 13–22, 10.1016/j.jcat.2014.10.014.

(S21) da Silva, L. F.; Maia, L. J. Q.; Bernardi, M. I. B.; Andrés, J. A.; Mastelaro, V. R. An Improved Method for Preparation of $SrTiO_3$ Nanoparticles. *Mater. Chem. Phys.* 2011, *125*, 168–173, 10.1016/j.matchemphys.2010.09.001.

(S22) Wei, X.; Xu, G.; Ren, Z.; Xu, C.; Shen, G.; Han, G. PVA-Assisted Hydrothermal Synthesis of $SrTiO_3$ Nanoparticles with Enhanced Photocatalytic Activity for Degradation of RhB. *J. Am. Ceram. Soc.* 2008, *91*, 3795–3799, 10.1111/j.1551-2916.2008.02716.x.

(S23) Sulaeman, U.; Yin, S.; Sato, T. Solvothermal Synthesis and Photocatalytic Properties of Chromium-Doped $SrTiO_3$ Nanoparticles. *Appl. Catal. B Environ.* 2011, *105*, 206–210, 10.1016/j.apcatb.2011.04.017.

(S24) Abdi, M.; Mahdikhah, V.; Sheibani, S. Visible Light Photocatalytic Performance of La-Fe Co-Doped $SrTiO_3$ perovskite powder. *Opt. Mater.* 2020, *102*, 109803, 10.1016/j.optmat.2020.109803.

(S25) Faisal, M.; Harraz, F. A.; Ismail, A. A.; El-Toni, A. M.; Al-Sayari, S. A.; Al-Hajry, A.; Al-Assiri, M. S. Polythiophene/Mesoporous $SrTiO_3$ Nanocomposites with Enhanced Photocatalytic Activity under Visible Light. *Sep. Purif. Technol.* 2018, *190*, 33–44, 10.1016/j.seppur.2017.08.037.

(S26) Iwanami, K.; Choi, J.-C.; Lu, B.; Sakakura, T.; Yasuda, H. Remarkable Acceleration of Cyanosilylation by the Mesoporous Al-MCM-41 Catalyst. *Chem. Commun.* 2008, No. 8, 1002–1004, 10.1039/b718462j.

(S27) Garnes-Portolés, F.; Rivero-Crespo, M. Á.; Leyva-Pérez, A. Nanoceria as a Recyclable Catalyst/Support for the Cyanosilylation of Ketones and Alcohol Oxidation in Cascade. *J. Catal.* 2020, *392*, 21–28, 10.1016/j.jcat.2020.09.032.

(S28) Ogasawara, Y.; Uchida, S.; Yamaguchi, K.; Mizuno, N. A Tin-Tungsten Mixed Oxide as an Efficient Heterogeneous Catalyst for C–C Bond-Forming Reactions. *Chem. – Eur. J.* 2009, *15*, 4343–4349, 10.1002/chem.200802536.

(S29) Higuchi, K.; Onaka, M.; Izumi, Y. Solid Acid and Base-Catalyzed Cyanosilylation of Carbonyl Compounds with Cyanotrimethylsilane. *Bull. Chem. Soc. Jpn.* 1993, *66*, 2016–2032, 10.1246/bcsj.66.2016.

(S30) Onaka, M.; Higuchi, K.; Sugishita, K.; Izumi, Y. Efficient Solid Catalyst Systems for Cyanosilylation of Carbonyl Compounds with Cyanotrimethylsilane. *Chem. Lett.* 1989, *18*, 1393–1396, 10.1246/cl.1989.1393.

(S31) Choudary, B. M.; Narender, N.; Bhuma, V. Calcined $MgAICO_3$-HT Catalysed Cyanosilylation of Carbonyl Compounds and Nucleophilic Ring Opening of Oxiranes Using TMSCN. *Synth. Commun.* 1995, *25*, 2829–2836, 10.1080/00397919508011830.

(S32) Atashkar, B.; Rostami, A.; Tahmasbi, B. Magnetic Nanoparticle-Supported Guanidine as a Highly Recyclable and Efficient Nanocatalyst for the Cyanosilylation of Carbonyl Compounds. *Catal. Sci. Technol.* 2013, *3*, 2140–2146, 10.1039/c3cy00190c.

(S33) Yamaguchi, K.; Imago, T.; Ogasawara, Y.; Kasai, J.; Kotani, M.; Mizuno, N. An Immobilized Organocatalyst for Cyanosilylation and Epoxidation. *Adv. Synth. Catal.* 2006, *348*, 1516–1520, 10.1002/adsc.200606124.

(S34) Martín, S.; Porcar, R.; Peris, E.; Burguete, M. I.; García-Verdugo, E.; Luis, S. V. Supported Ionic Liquid-like Phases as Organocatalysts for the Solvent-Free Cyanosilylation of Carbonyl Compounds: From Batch to Continuous Flow Process. *Green Chem.* 2014, *16*, 1639–1647, 10.1039/c3gc42238k.

(S35) Kantam, M. L.; Sreekanth, P.; Santhi, P. L. Cyanosilylation of Carbonyl Compounds Catalyzed by a Diamino-Functionalised Mesoporous Catalyst. *Green Chem.* 2000, *2*, 47–48, 10.1039/a908758c.

(S36) Curini, M.; Epifano, F.; Marcotullio, M. C.; Rosati, O.; Rossi, M. Potassium Exchanged Zirconium Hydrogen Phosphate as Heterogeneous Catalyst in Cyanosilylation of Carbonyl Compounds. *Synlett* 1999, *1999*, 315–316, 10.1055/s-1999-2597.

(S37) Jia, Y.; Zhao, S.; Song, Y.-F. The Application of Spontaneous Flocculation for the Preparation of Lanthanide-Containing Polyoxometalates Intercalated Layered Double Hydroxides: Highly Efficient Heterogeneous Catalysts for Cyanosilylation. *Appl. Catal. Gen.* 2014, *487*, 172–180, 10.1016/j.apcata.2014.09.005.

(S38) Rojas-Buzo, S.; García-García, P.; Corma, A. Remarkable Acceleration of Benzimidazole Synthesis and Cyanosilylation Reactions in a Supramolecular Solid Catalyst. *ChemCatChem* 2017, *9*, 997–1004, 10.1002/cctc.201601407.

(S39) Gomez, G. E.; D'vries, R. F.; Lionello, D. F.; Aguirre-Díaz, L. M.; Spinosa, M.; Costa, C. S.; Fuertes, M. C.; Pizarro, R. A.; Kaczmarek, A. M.; Ellena, J.; et al. Exploring Physical and Chemical Properties in New Multifunctional Indium-, Bismuth-, and Zinc-Based 1D and 2D Coordination Polymers. *Dalton Trans.* 2018, *47*, 1808–1818, 10.1039/C7DT04287F.
